# Influence of Land Development on Holocene *Porites* Coral Calcification at Nagura Bay, Ishigaki Island, Japan

**DOI:** 10.1371/journal.pone.0088790

**Published:** 2014-02-24

**Authors:** Kohki Sowa, Tsuyoshi Watanabe, Hironobu Kan, Hiroya Yamano

**Affiliations:** 1 Department of Natural History Sciences, Hokkaido University, Sapporo, Japan; 2 Graduate School of Education/Graduate School of Natural Sciences, Okayama University, Okayama, Japan; 3 Center for Environmental Biology and Ecosystem Studies, National Institute for Environmental Studies (NIES), Tsukuba, Japan; New England Aquarium, United States of America

## Abstract

To evaluate the relationships between coral calcification, thermal stress, and sedimentation and eutrophication linked to human impact (hereafter referred to as “land development”) by river discharge, we analyzed growth characteristics in the context of a paleoenvironment that was reconstructed from geochemical signals in modern and fossil (1.2 cal kyr BP and 3.5 cal kyr BP, respectively) massive *Porites* corals from Nagura Bay (“Nagura”) and from modern *Porites* corals from the estuary of the Todoroki River, Shiraho Reef (“Todoroki”). Both sites are on Ishigaki Island, Japan, and Nagura is located approximately 12 km west of Todoroki. At Nagura, the individual corals provide time windows of 13 (modern), 10 (1.2 cal kyr BP), and 38 yr in length (3.5 cal kyr BP). Here, we present the coral annual calcification for Nagura and Todoroki, and (bi) monthly resolved records of Sr/Ca (a proxy of sea surface temperature (SST)) and Ba/Ca (a proxy of sedimentation and nutrients related to land development) for Nagura. At Nagura, the winter SST was cooler by 2.8°C in the 1.2 cal kyr BP, and the annual and winter SSTs in the 3.5 cal kyr BP were cooler by 2.6°C and 4.6°C, respectively. The annual periodicity of Ba/Ca in modern coral is linked to river discharge and is associated with land development including sugar cane cultivation. Modern coral calcification also has declined with SST warming and increasing Ba/Ca peaks in winter. However, calcification of fossil corals does not appear to have been influenced by variations in Sr/Ca and Ba/Ca. Modern coral growth characteristics at Nagura and Todoroki indicate that coral growth is both spatially and temporally influenced by river discharge and land development. At Nagura, our findings suggest that land development induces negative thermal sensitivity for calcification in winter due to sugar cane harvest, which is a specifically modern phenomenon.

## Introduction

Coral calcification is an important barometer of the physiological response of coral to changes in abiotic environmental factors, such as sea surface temperature (SST), sediment discharge, nutrients, and aragonite saturation state [1]. One of the most prominent negative impacts on coral calcification is coral bleaching, which occurs as a result of collapsing relationships between coral hosts and their resident photosynthetic dinoflagellates [2]. SST warming is often cited as the main cause of large-scale coral bleaching [3]. To predict bleaching events, either degree heating months (DHM) or degree heating weeks (DHW) can be used as a proxy of cumulative heat stress; these values are calculated based on the monthly or weekly averaged SST, respectively [4]–[6]. However, prior to 1979, the Florida Keys and Mesoamerican Reef exhibited rare or even no bleaching events, even during high DHM periods [7]–[9]. These results imply that coral bleaching in response to thermal stress is a modern phenomenon, raising the question of the cause of recent coral bleaching events.

One suggested reason is increasing oceanic nutrient levels as a result of land development [9], [10]. Based on *in situ* nutrient data and model analysis at the Great Barrier Reef [10], poor water quality in coral reefs as a result of increasing land development likely results in coral with decreased thermal tolerance. To verify this hypothesis, estimations of the influence of long-term nutrient exposure on coral growth are needed. Recent global and local environmental changes are the result of both natural variation and post-industrial era human activity. Hence, knowledge of coral growth from the pre-industrial era should provide useful information on the respect to the natural conditions in coral reefs.

Massive coral skeletons are useful for providing long-term (several hundred years or more in length) retrospective data of coral growth. Corals grow by depositing an aragonitic skeleton that exhibits high- and low-density bands within 1 yr [11]. This density banding provides historical information about mean annual skeletal density (average bulk density; g cm^−3^) and annual extension rate (cm yr^−1^), which can be multiplied to obtain the annual calcification rate (g cm^−2^ yr^−1^) [12]. These growth parameters have been successfully analyzed previously by non-destructive methods, including X-radiography, computed tomography, and γ-densitometry [13]–[15].

Coral sclerochronology has been used to report a possible reduction of thermal tolerance threshold in the coral extension rate of massive *Montastraea faveolata* in the Caribbean Sea [9]. The effects of chronic local stressors as a result of the human population were better predictors for explaining the recent reductions of the coral extension rate than DHM. However, at least two problems have been identified: (1) no direct comparisons between coral growth and environmental data exist, and (2) the analysis were performed in the modern era, in which land development was underway.

Geochemical signals, such as isotopic or elemental profiles (e.g., δ^18^O or Sr/Ca and Ba/Ca ratios) in coral skeletons provide in a quantitative manner both precise chronology and past sea surface environmental changes. Many coral growth studies have adopted the back counting of coral density bands to determine chronology, in which a pair of high- and low-density bands is assumed to represent 1 yr [13]–[21]. However, *Porites* corals sometimes develop several high-density bands in 1 year [22], and differences in the deposition timing of high/low density bands have been reported among coral genders [23]. Further, chronology developed by the simple back counting of density bands also incurs chronological error (± several years) [24]. However, geochemical signals can extract precise chronology of coral growth with errors on the order of a year.

Geochemical signals in coral skeletons can also be used to reconstruct a paleoenvironment, including SSTs and river discharge at sites where no instrumental records exist. The Sr/Ca ratio in the coral skeleton can provide a robust proxy and is commonly used as a paleo-thermometer (e.g., [25]–[27]). Similarly, Ba in seawater is closely associated with upwelling, river discharge, and terrestrial sediment input, which can be reconstructed from coral Ba/Ca ratios [28]–[38]. Thus, the geochemical signals in coral skeletons enable us to analyze the relationships between coral growth and past environmental changes.

This study aimed to evaluate whether the relationships between coral calcification rate and thermal stress are specifically modern characteristics using Holocene modern and fossil corals collected at the same site. First, we analyzed the skeletal growth characteristics of massive *Porites* corals. Second, we measured the geochemical signals (the Sr/Ca and Ba/Ca ratios) in modern and fossil coral skeletons to reconstruct paleoenvironmental data. Sr/Ca thermometry calibrated by modern coral provided Holocene seasonal SSTs. Ba/Ca ratios were measured to evaluate the nutrient and sediment supplies from river discharges. We then compare and discuss the relationships between coral calcification and environmental abiotic parameters, which were reconstructed from the Sr/Ca and Ba/Ca ratios in the coral skeletons.

## Materials and Methods

### Setting of the study area

**Figure 1:**
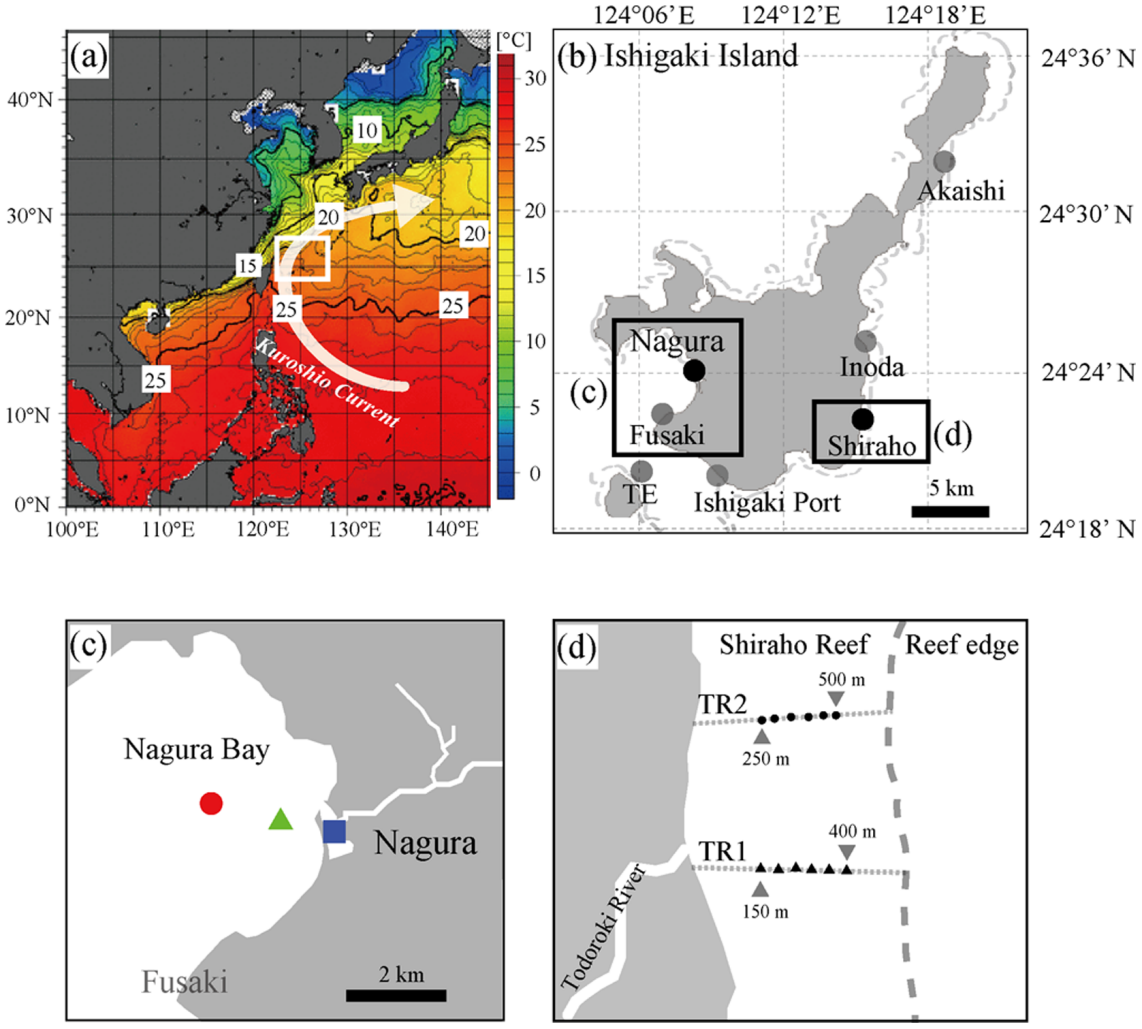
Location of coral sampling site and Feb. 2012 averaged SST from the Japan Meteorological Agency (http://www.jma.go.jp/jma/indexe.html). (a). White square denotes the sampling site. Core A7 [41], [68], IODP 1202B [69] and Kume Island [70] are in white square. White arrow shows the Kuroshio Current. (b) Enlarged view of the region marked by the white square in (a), Ishigaki Island. Fusaki, Akaishi, Inoda, and TE are reference sites in this study [59], [88]. (c) Enlarged view of the region marked by the left black square in (b), Nagura Bay. Red circle indicates the modern coral sampling point. Green triangle and blue square indicate the sample collection sites in 1.2 kyr BP and 3.5 kyr BP fossil coral, respectively. Dotted line shows the boundary between the Shiraho Reef (left) and Reef edge (right). (d) Enlarged view of the region marked by the right black square in (b), Todoroki Estuary. Small black circles and triangles indicate sampling points with 50-m intervals of line transects (TR1 and TR2), respectively.

Modern and fossil corals were collected in August 2009 from Nagura Bay (“Nagura”) on the west side of Ishigaki Island, and modern corals were collected in February 2012 from the estuary of the Todoroki River (“Todoroki”), Shiraho Reef, Ishigaki Island, Japan (Figure 1 (a) and (b)). The atmospheric circulation and oceanographic setting of the study sites are characterized by the Kuroshio Current, which contains warm and salty water, and the East Asia Monsoon (EAM), which consists of the East Asia Summer and Winter Monsoons. On the geological time scale, the EAM is linked to a high-latitude Northern Hemisphere climate [39]–[41]. The East Asia Winter Monsoon is influenced by the Siberian High and brings cold winds. The prominent wind direction is south in summer and north in winter.

The Nagura River is the primary contributor of fresh water to Nagura Bay on Ishigaki Island (Figure 1 (c)). The river is 4.6 km long with a catchment area of 16.1 km^2^, and estuarine tidal flats and mangrove forests cover 157 ha at the mouth of the river. Twenty percent of the watershed has been developed for agricultural purposes within the last three decades [42]. Red soil associated with land development flows from the land area, increasing nutrient levels and sedimentation and influencing seawater ecosystems in Nagura Bay [42]. In the Shiraho Reef, fresh water is introduced by the Todoroki River (Figure 1 (d)) and the dominant coral reef current flows northward from the river mouth [43]. This river water transports red soil and nutrients originating from the watershed, which is dominated by agricultural land.

Direct measurements of the annual and monthly SST data were obtained from the World Wildlife Fund Coral Reef Conservation and Research Center from 2002 to 2009, taken at Shiraho Reef (24°22′02.7″N, 124°15′32.3″E), and from the Japan Meteorological Agency from 1914 to 2006 (JMA; http://www.jma.go.jp/jma/indexe.html), measured at Ishigaki Port (24°20′N, 124°08′E) (Figure S1). The monthly SST of Shiraho Reef is generally warmer than that of Ishigaki Port in Ishigaki Island (Figure S2). The averaged SST difference between Shiraho Reef and Ishigaki Port was approximately 0.5°C from 2002 to 2006 (Figure S2). The satellite-derived monthly SST time series from 2002 to 2006 at 1° × 1° (24.5°N, 124.5°E) was derived from Integrated Global Ocean Services System Products Bulletin (http://iridl.ldeo.columbia.edu/SOURCES/.IGOSS/) [44]. We converted the SSTs from Shiraho Reef and referred to those at Ishigaki Port for the overlapping period from July 2002 to February 2006 (Figure S3). Direct measurements of air temperature (°C), global solar radiation (MJ m^−2^), precipitation (mm), wind speed (m s^−1^), and the number of typhoons achieving landfall on Ishigaki Island from June to September were obtained from the JMA (Figures S1 and S2). The monthly average SST varies from 21.1 to 29.9°C (average 25.3°C). From 1996 to 2008, the warmest monthly average SST was in July (28.9°C), and the coolest, in February (21.6°C). Similarly, the monthly average air temperature varies from 17.5 to 30.5°C (average 24.5°C). From 1996 to 2008, the warmest monthly average air temperature was in July (29.5°C), and the coolest, in January (18.9°C). Lastly, from 1996 to 2008, monthly insolation varied from 6.8 to 26.7 MJ m^−2^ (average 15.5 MJ m^−2^) and monthly precipitation varied from 11.5 to 826 mm (average 174 mm).

Instead of DHW [4], [5], [45], we applied DHM as an indicator of cumulative heat stress for the modern and fossil corals [6], [9], [19]. The DHM was calculated based on the annual sum of the difference between the average monthly SSTs exceeding the long-term maximum monthly mean, recorded from 1914 to 2009 at Ishigaki Port by the JMA.

### Coral preparation

A modern massive *Porites* sp. coral colony measuring about 20 cm in diameter was sampled from the sub tidal area several meters below the low tide position at Nagura Bay (Figure 1 (c)). A 2.33 m drilling core was obtained from the Holocene reef at Nagura Bay at 1.8 m below mean sea level using a diver-operated submersible KAN-type hydraulic drill (GeoAct Co. Ltd, Kitami, Japan). The top 38 cm of the core consisted of five pieces of fossil *Porites* sp. (Figure 1 (c)), the uppermost piece of which was used for this study. A fossil microatoll of *Porites* sp. coral was collected from the estuary of the Nagura River (Figure 1 (c)). We also collected massive modern *Porites* coral colonies that were within 30 cm in diameter from the sub tidal area several meters below the low tide position near Todoroki, Shiraho Reef, along approximately 50 m intervals of line transects from inshore to offshore (TR1 and TR2) on August 2009 (n = 6) and on February 2012 (n = 22) to examine the influence of factors such as red soil and nutrient loading from land development (Figure 1 (d)). The permits to collect the samples were issued by Okinawa Prefecture and Ministry of the Environment.

To prepare coral slabs from coral cores and colonies, slices measuring approximately 2–5 mm in thickness were cut under water flow along the axis of main growth using a rock saw with a diamond-tipped blade, and slices were planed uniformly. Each slice was rinsed multiple times with Milli-Q water in a sonicator, dried for several days at approximately 40°C in a laboratory oven, and X-radiographed using TATSCAN-X1 with a digital imaging intensifier X-ray camera. The exposure settings were 29.6–32.5 kV and 2.02 mA.

**Figure 2:**
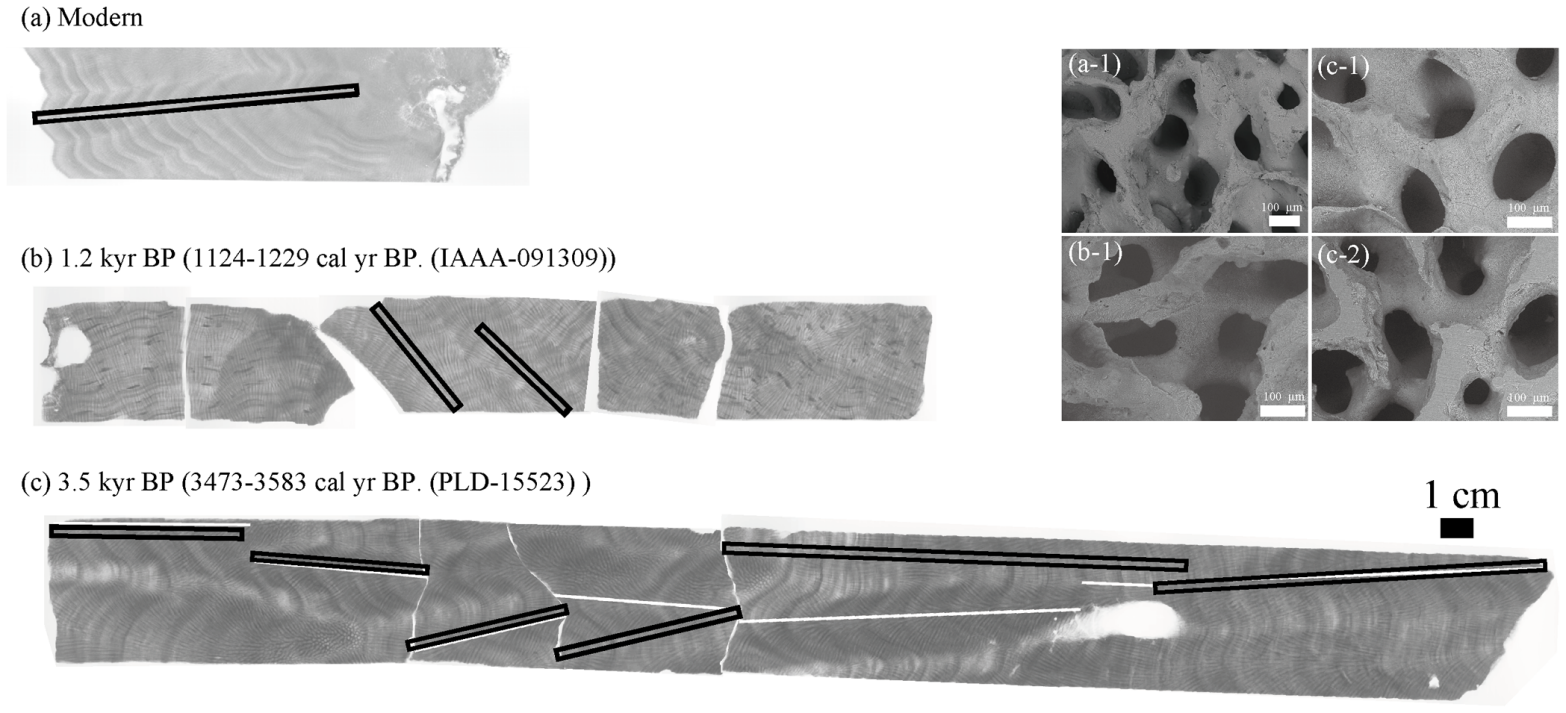
Positive X-radiograph and scanning electron microscope (SEM) images of (a) modern, (b) 1.2 kyr BP, and (c) 3.5 kyr BP *Porites* coral slabs. Black lines in (a) and (b) indicate the position of the micro-sampling and growth analysis transects. In (c), white and black lines indicate the micro-sampling and growth analysis line, respectively. The SEM images of (a-1) modern, (b-1) 1.2 kyr BP and (c-1) top growth and (c-2) bottom growth areas of 3.5 kyr BP slabs.

Corals collected in Nagura Bay in this study were investigated for possible diagenetic alternation and secondary mineral deposits in their skeleton by field-emission scanning electron microscopy (FE-SEM) (TM1000; Hitachi High-Technologies Corporation) and X-ray diffraction (XRD) analysis, both applied to skeletal fragments at intervals every 10 cm along the coral maximum growth line (Figure 2).

### Geochemical analysis (^14^C, Sr/Ca and Ba/Ca ratios)

The conventional radiocarbon ages of the corals were analyzed by accelerator mass spectrometry at the Institute of Accelerator Analysis Ltd. and at Paleo Labo Co., Ltd.

The Sr/Ca and Ba/Ca coral analyses were performed with inductively coupled plasma-atomic emission spectrometry (iCAP 6300 ICP Spectrometer; Thermo Scientific) using an auto sampler (CETAC ASX-260) at Hokkaido University. The modern and fossil coral samples were milled continuously at an average interval from approximately 0.5–1 mm along the corallite growth direction and were then transferred to individual holders in preparation for analysis of the metals. High-pressure air cleaning was applied after each sub-sample to avoid cross-contamination. The sampling resolution was equivalent to approximately 6 samples per annual growth increment for the modern corals and approximately 12 samples per annual growth increment for the fossil corals collected at Nagura. Approximately 0.2 mg of each coral sample was powdered and dissolved in about 3 mL of 4 mol L^−1^ high-purity HNO_3_ diluted with ultrapure Milli-Q water. Calibrations of the four gravimetric standard solutions yielded high correlation coefficients (*r^2^*) of >0.999 for Sr and Ca. A reference solution of JCp-1 [46], which was gravimetrically matched in concentration and matrix to the average of the coral sample solutions, was measured at 5 sample intervals to correct for instrumental drift. Based on replicate measurements of the reference solution for the coral analyses, the respective external precision values (relative uncertainties) were 0.13% and 0.30% for the Sr/Ca and Ba/Ca determinations, respectively (n = 48).

### Chronology development

Conventional radiocarbon ages for fossil corals were obtained after correcting for isotope fractionation (1σ). To correct for the ocean reservoir effect, a local calibration value (ΔR = 35±25 ^14^C yr) was applied, as in a previous report [47]. The calibrated ages of the coral samples were calculated using Marine09 and the CALIB 6.1 program (CALIB Radiocarbon Calibration; http://calib.qub.ac.uk/calib/).

The age models for the corals were based on both the annual density-band patterns in the X-radiographs and the cycles in Sr/Ca. We assumed that the timing of the annual SST cycle did not change during the investigated period. Therefore, the maximum and minimum Sr/Ca ratios in a given year were assigned to the minimum (July) and maximum (February) SST values in the year, respectively. Subsequently, the other Sr/Ca values were plotted by linear interpolation between the fixed points.

### Growth analysis

In the X-radiographs, the coral growth was calculated using the mean annual skeletal density as the average skeletal density between adjacent annual skeletal density maxima or minima (winter); the mean annual extension rate was calculated as the linear distance between adjacent annual skeletal density maxima or minima (winter) [48], [49]. For the corals collected from Nagura, periodical Sr/Ca calibrated the deposition timing of their skeleton. It was supposed that the high-density band was deposited in the winter season for the corals collected from Todoroki [50]. To correct the effects of the inverse square law and heel effect [51], we used the CoreCal 2 program [50]. An aluminum bar with the same thickness as the coral slab was included on each digital X-radiograph, placed along the X (horizontal) and Y (vertical) axes of the X-ray machine, as well as an aragonitic step wedge built of blocks cut from a shell of the giant clam *Hippopus hippopus* as standards for analyzing coral skeletal density. The skeletal density of the giant clam was 2.85 g cm^−3^ and synthesized standard uncertainty was 0.00223 g cm^−3^. The averaged observed density (OD; the gray scale value of pixels; 0–255) was used to obtain factors that corrected for the effects at any distance on the X-radiography. X and Y lines resulted in an X-radiography when OD values for aluminum bars were adjusted by CoreCal2. The OD was analyzed using the software Image J 1.42q (Wayne Rasband, National Institutes of Health, USA). The corrected digital X-radiographies were used to measure the skeletal density along the vertical growth axis. The thickness of coral slices was measured 10 times along the growth axis using a set of calipers (±0.01 mm) and in all areas used for bulk skeletal density analysis. The analyzed ODs were then converted to logarithmic ODs, which were used to calculate the skeletal density. The uncertainties of skeletal density were analyzed and all uncertainties were <0.05 g cm^−3^ (see [50] for details).

### Statistics

In our statistical analyses, we investigated the influence of environmental factors (SST, global solar radiation, precipitation, DHM, and Ba/Ca ratio) and their long-term trends as they relate to coral growth and the periodicity of the geochemical signals for the coral collected in Nagura Bay. Several potential abiotic predictors of the modern and fossil coral calcification rates in Nagura Bay were examined, including annual SST, precipitation, insolation, minimum SST, DHM, and annual and/or peak Ba/Ca ratios. The models were explored using a generalized linear model (GLM) with Gaussian distribution. Several models tested the relationships between skeletal density and extension rate in massive and small *Porites*, depth, and distance from the coast for the coral collected from Todoroki. The models were explored using GLM with Gamma distribution and a log link.

To select the predictors in the statistical models of each coral growth, various measurements of the goodness of fit were applied to identify the best prediction model. These measures included the Akaike Information Criterion (AIC), the best of which was selected. For a given model, the AIC was calculated as AIC = 2l +2K, where l is the maximized log likelihood and K is the number of parameters in the model.

Multicollinearity among predictor variables may have adverse effects on the coefficients estimated in a multiple regression. The variance inflation factors (VIF) were computed to detect the existence of multi-collinearity in our data. A cut-off value of VIF = 10 was adopted.

The long-term coral growth trends were explored using a generalized state-space model for a time series analysis. Coral growth was not considered as an individual parameter for each year [17], [52], [53]. A stochastic local linear trend model was applied, supposing that coral growth would correlate over time. The coral calcification rate (*Y_t_*) includes a time-varying slope in the dynamics for *µ_t_* with uncorrelated errors *υ_t_*, *ω_t,1_*, and *ω_t,2_*.
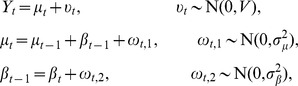



To estimate the uncertainty of the unknown parameters, we used Bayesian statistics with Markov Chain Monte Carlo (MCMC) methods. The gamma prior mean and variance of observation were 1 and 1,000, respectively. The gamma prior mean(s) and variance(s) of the evolutionary precision(s) were 1 and 1,000, respectively. The generated sample size was 50,000 with *thin = 2*, and the first approximately 5,000–20,000 saved iterations were considered as burn-in data (modern coral calcification rates of *μ*, *σ^2^_μ_*, *σ^2^_β_* were 15,000 and *β* was 10,000; the 1.2 kyr BP coral calcification rates of *μ*, *β*, *σ^2^_μ_*, *σ^2^_β_* were 5,000; the 3.5 kyr BP coral calcification rate of *μ*, *β*, *σ^2^_μ_*, *σ^2^_β_* were 10,000). Parameter convergences were verified by using Geweke’s convergence diagnostic [54]. These analyses were performed using the AnalySeries 2.0 software, dlm 1.1–2 and boa 1.1.7–2 of R 2.14.1 [55]–[58].

To determine the origin of the Ba/Ca variation in the modern and fossil corals, we analyzed the periodicity of these measurements. The Blackman-Tukey method and cross-spectral analysis were applied with coherency exceeding the 90% confidence interval using AnalySeries. Prior to the spectral analyses, the time series were detrended.

## Results

### Preservation of the coral skeletons and ^14^C-dating of the corals

The X-radiographs of individual coral slabs revealed clear annual-density banding patterns (Figure 2). We selected analysis lines that did not include changing density patterns, a typical characteristic of diagenetic alteration in coral skeletons at Nagura [59]. XRD analysis did not show clear calcite or BaSO_4_ peaks. SEM images revealed areas that were free of secondary overgrowth and had no clear dissolution areas along the lines of analysis. Hence, both the modern and fossil corals had pristine aragonitic skeletons.

The conventional radiocarbon ages of the fossil corals were 1124–1229 cal yr BP. (IAAA-091309) (hereafter abbreviated as “1.2 kyr BP”) and 3473–3583 cal yr BP. (PLD-15523) (hereafter “3.5 kyr BP”).

### Coral growth characteristics

**Figure 3:**
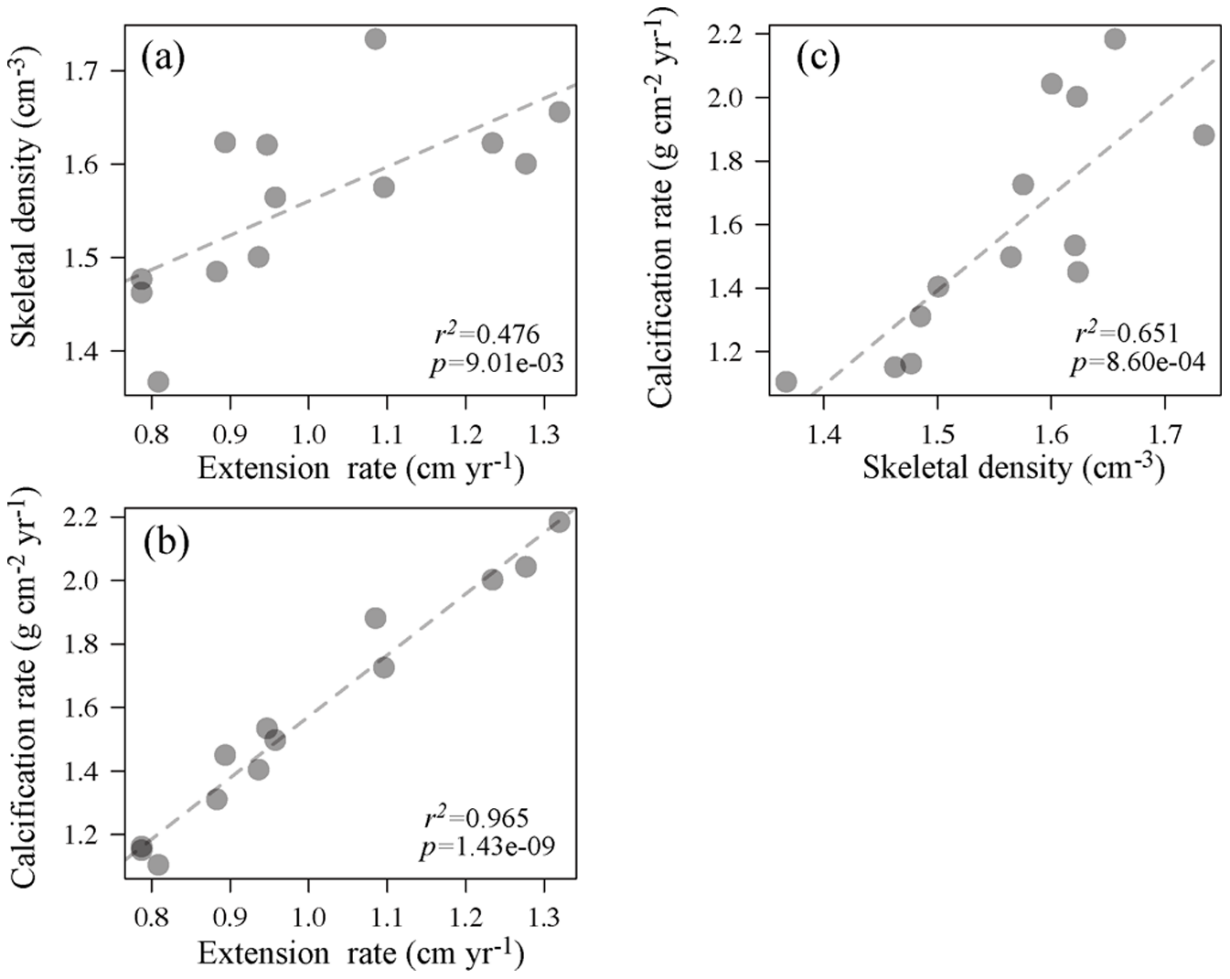
Scatter diagrams of modern coral growth data from Nagura Bay. (a) Skeletal density vs. extension rate, (b) calcification rate vs. extension rate, (c) calcification rate vs. skeletal density. Regression lines are shown where there is a statistically significant link.

Significant variation between the one modern and two fossil *Porites* colonies at Nagura was found for all three growth parameters (skeletal density, extension rate, and calcification rate). The extension rate and skeletal density were positively correlated in modern coral (*r* ^2^ *= *0.476, *p = *9.01e-03; Figure 3). However, in the fossil corals, the extension rate was not related to skeletal density. We adopted coral calcification as a typical parameter for coral growth at Nagura.

**Figure 4:**
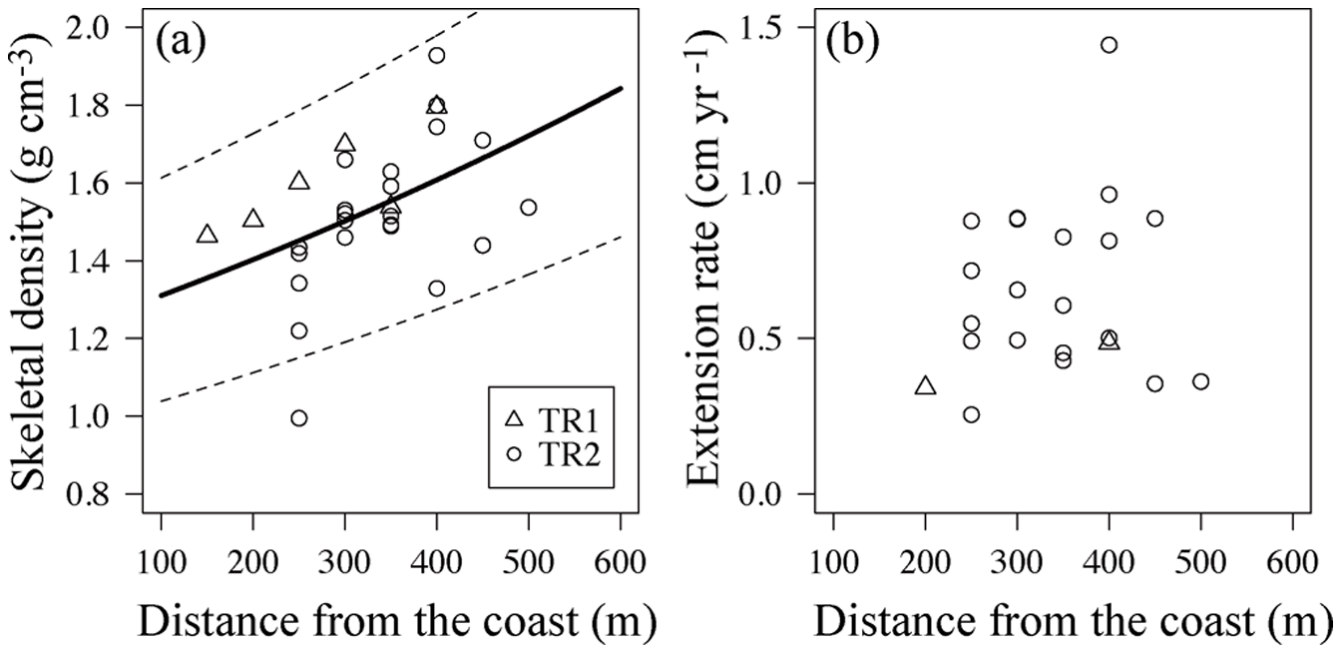
Relationships between (a) skeletal density and (b) extension rate and distance from the coast at TR1 (triangle) and TR2 (circles) in the Todoroki Estuary. Solid and dotted lines indicate the mean curve and 95% prediction intervals, respectively.

At Todoroki (TR1 and TR2), skeletal density increased with increasing distance from the coast (Figure 4(a), Table 1). However, no parameters were selected for variation of extension rate (Figure 4 (b), Table 1).

**Table 1:** Statistical model parameter estimates of modern corals at the estuary of the Todoroki River (TR1 and TR2).

Site		Model	AIC
**TR1 and TR2**	**1**	**Density∼Distance**	**−13.6**
	2	Density∼Distance+Depth	−10.6
	3	Density∼none	−9.8
**TR1 and TR2**	**1**	**Extension rate∼none**	**4.3**
	2	Extension rate∼Distance	5.4
	3	Extension rate∼Depth	7.1

Bold text indicates the selected models.

**Figure 5:**
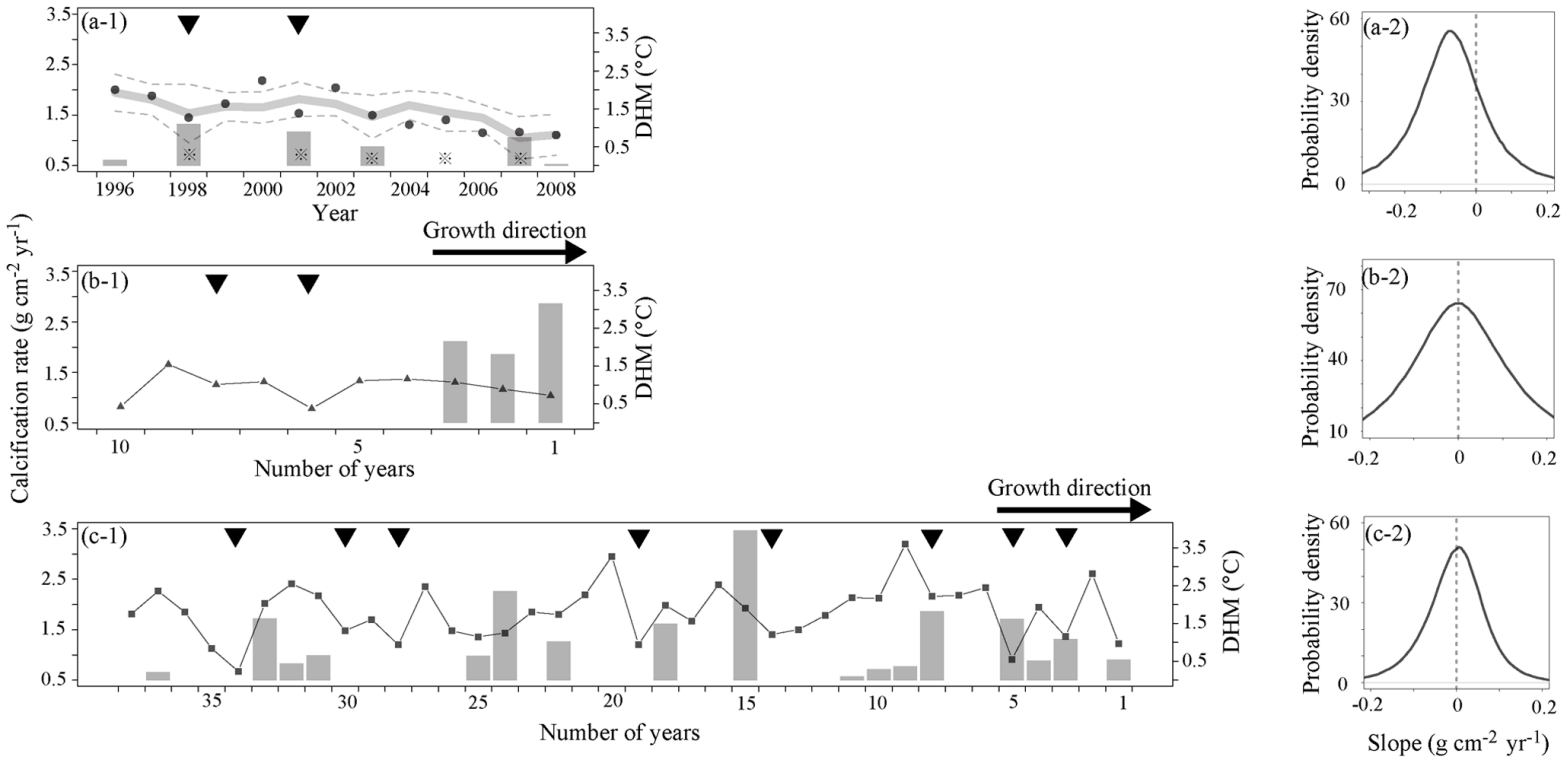
Long term variation and slope of calcification rate in (a-1, a-2) modern, (b-1, b-2) 1.2 kyr BP, and (c-1, c-3) 3.5 kyr BP corals at Nagura. Faint gray and gray dotted lines in (a-1) denote the model prediction (see Table 2 for more details) and 95% confidence line. Gray vertical bars in (a-1), (b-1), and (c-1) indicate degree heating months (DHM). Triangles denote the <−22.9% reduction from the previous year and increasing trend in the next year. Asterisk indicates the observed bleaching events based on [114].

Estimate (posterior distribution means) of long-term trends (slopes) were −0.70 g cm^−2^ 10 yr^−1^ (76.7% Bayesian credible interval of slope was less than 0) in modern coral, +0.047 g cm^−2^ 10 yr^−1^ (51.4% Bayesian credible interval of slope was more than 0) in the 1.2 kyr BP coral, and −0.008 g cm^−2^ 10 yr^−1^ (50.4% Bayesian credible interval of slope was less than 0) in the 3.5 kyr BP coral (Figure 5). The fossil corals did not exhibit clear decreasing or increasing calcification rate trends.

We compared the modern coral extension rate we found in our study at the Nagura with that reported by Omata et al. [59] to test whether this rate is specimen specific (Figure S4). We reanalyzed average annual extension anomaly (%) in three coral cores (TE 1, 3, and 4) from the TE site (24°20′20.58″N, 124°06′3.4″E), the closest site to Nagura. Although the coral cores revealed the extension rate for 1979–2000, we focused only on the more cores from 1990 to 2000. Figure S4 shows the relationship between extension rate and year in massive *Porites* sp. corals from the 2 sites for 1990–2008, which showed a significantly similar decreasing trend. This indicates that the coral growth trend around Nagura Bay was not specimen specific.

### Coral Sr/Ca- SST thermometry

Bimonthly SST regression slopes from the modern *Porites* sp. coral were assessed for the period 1996–2008. The regression equation for the Sr/Ca-SST calibration is given in Figure S5 and below:
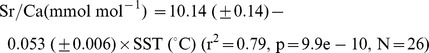



The uncertainty in the coral Sr/Ca-based SST was calculated by combining the uncertainties of the Sr/Ca ratio and the thermometry regression. Taken together, the maximum uncertainty for the reconstructed SST record for the fossil corals was approximately ±1.05°C (*k = *1) (Figure S5).

**Figure 6:**
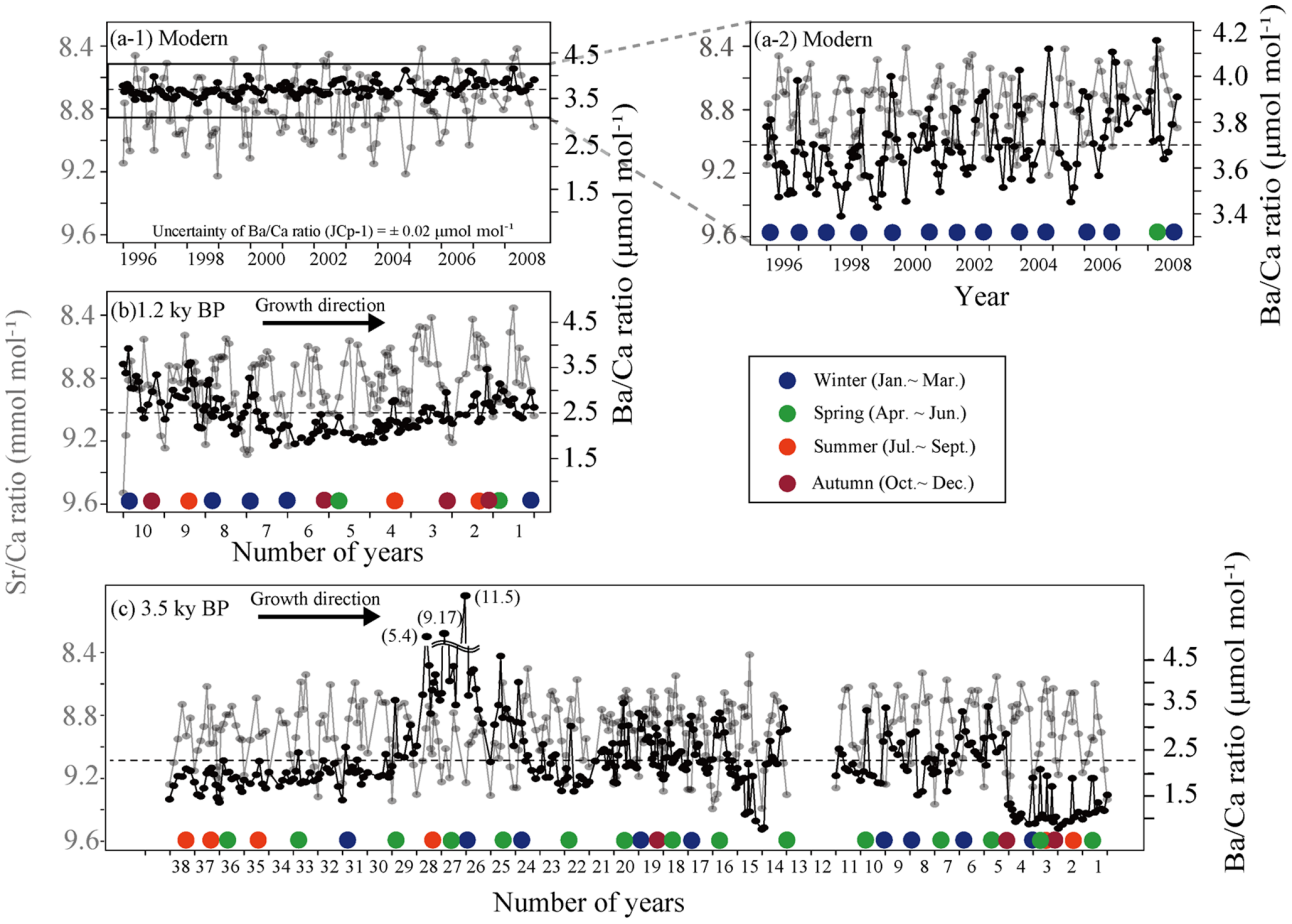
Plots of Sr/Ca (gray line) and Ba/Ca (black line) ratios as time series for one modern (a-1 and a-2), and two fossil (b and c) *Porites* corals. (a-2) is the magnification of (a-1) at Nagura Bay. Color circles indicate high Ba/Ca. Dashed horizontal lines indicate the average Ba/Ca ratio in each coral.

The Sr/Ca records in the modern coral showed distinct annual cycles, which correlated to annual density-banding patterns in the skeletons (Figure 6). The modern coral Sr/Ca ratios ranged from 8.60 to 8.99 mmol mol^−1^ (average value 8.78 mmol mol^−1^). The 1.2 kyr BP coral values ranged from 8.52 to 9.20 mmol mol^−1^ (average value 8.83 mmol mol^−1^). The 3.5 kyr BP coral values ranged from 8.64 to 9.24 mmol mol^−1^ (average value 8.93 mmol mol^−1^).

**Figure 7:**
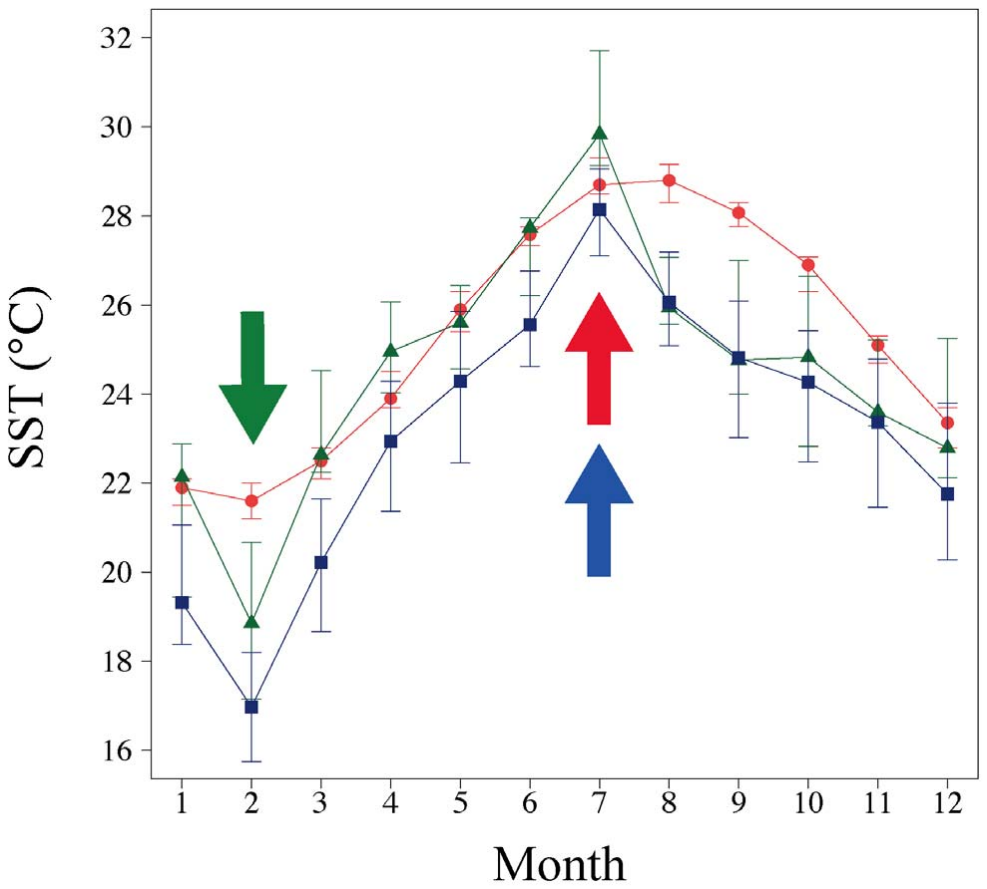
Interquartile range plot of monthly seasonal variations of instrument and reconstructed SSTs from corals collected at Nagura. Red circles, green triangles, and blue squares indicate modern instrument data, 1.2-density bands formation in modern (red), 1.2 kyr BP (green), and 3.5 kyr BP (blue) corals, respectively.

The monthly maximum and minimum SSTs were established based on the original data of the yearly minimum and maximum Sr/Ca ratios respectively (Figure 7). The Sr/Ca ratios confirmed that the high-density bands were deposited in the highest SST season (summer) in the modern and 3.5 kyr BP corals and were deposited in the lowest SST season (winter) in the 1.2 kyr BP coral (Figure 7). In the 1.2 kyr BP corals, the average winter minimum SSTs (hereafter “winter SST”) was approximately 2.8°C cooler (18.8°C) than at present (21.6°C), and in the 3.5 kyr BP corals, winter SST was approximately 4.6°C cooler (17.0°C) than at present. However, in the 3.5 kyr BP corals, the average annual SST (hereafter “annual SST,”) was approximately 2.6°C cooler (22.7°C) than at present (25.3°C). The summer maximum SST (hereafter “summer SST”) values in modern, 1.2 kyr BP, and 3.5 kyr BP corals were 29.1°C, 30.4°C, and 28.1°C, respectively.

### Ba/Ca ratios in the coral skeletons

Figure 6 shows the Ba/Ca ratios versus growth time at bimonthly and monthly resolutions for the modern and fossil corals, respectively. The Ba/Ca values in the modern coral ranged from 3.39 to 4.16 µmol mol^−1^ (average value 3.70±0.054 µmol mol^−1^), while that of the 1.2 kyr BP coral ranged from 1.78 to 3.92 µmol mol^−1^ (average value 2.51±0.037 µmol mol^−1^), and in the 3.5 kyr BP coral, from 0.76 to 11.5 µmol mol^−1^ (average value 2.28±0.034 µmol mol^−1^).

The Ba/Ca ratio had a yearly periodicity that was either in phase with the SST for modern coral or lagged behind it by approximately 6 months (178°) (Figure S6). The Ba/Ca measurements positively correlated with the monthly SST values of the modern coral (Figure S6 (a)). However, the Ba/Ca ratio in the fossil corals had insignificant periodicity (Figures S6 (b) and (c)). These results indicated that a yearly periodicity of the Ba/Ca ratio is a prominent characteristic in modern coral.

The seasonal peaks of Ba/Ca occurred mainly in the winter for modern coral (92.9%), in the winter for 1.2 kyr BP coral (35.7%), and in the spring for 3.5 kyr BP coral (45.5%) (Figure S7). The winter peaks of Ba/Ca exhibit a significantly increasing trend for the modern coral (*r* ^2^ = 0.53, *p = *0.0046).

We estimated the seawater Ba concentration based on a distribution coefficient of 1.27 [34]. In the modern coral skeletons, Ba ranged from 26.6 nmol kg^−1^ in the summer to a high of 32.6 nmol kg^−1^ in the winter. In the 1.2 kyr BP fossil corals Ba ranged from 14.1 to 30.8 nmol kg^−1^ and in the 3.5 kyr BP fossil corals from 6.00 to 90.7 nmol kg^−1^.

**Figure 8:**
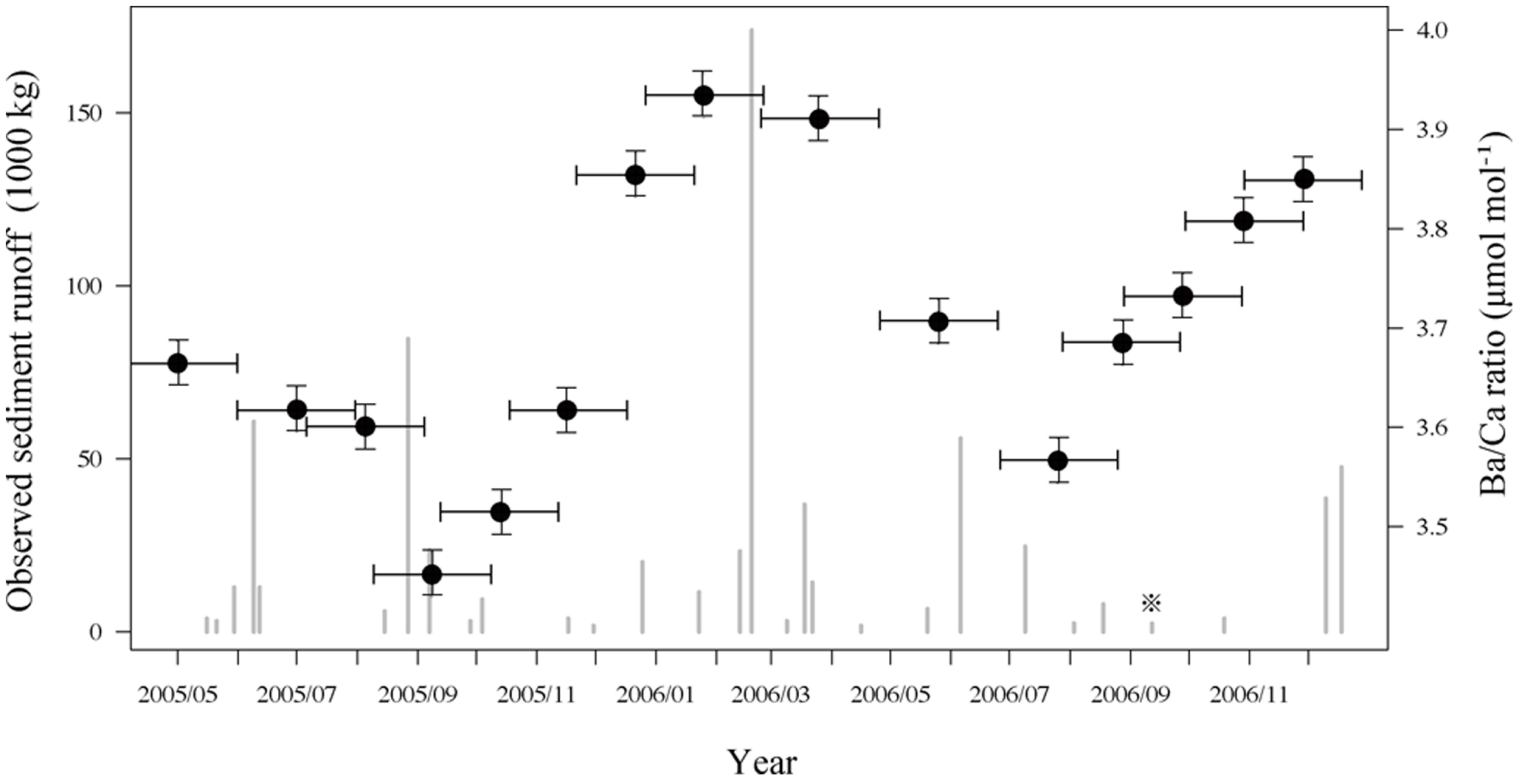
Comparison of sediment runoff (gray bars) and Ba/Ca ratio (black points) in modern coral from May 2005 to November 2006. Sediment runoff data is derived from [60]. A valid rainfall event was considered to be one that produced more than 12.7 mm of rain or one that produced more than 6.4 mm of rain in 15 min, after which rain stopped for more than 6 h. Two-month (maximum) error bars are plotted in each Ba/Ca ratio.

The observed sediment runoff [60] and coral Ba/Ca ratios are compared in Figure 8 for the dates from May 2005 to November 2006. These data indicated that high sediment runoff corresponded to coral Ba/Ca peaks occurring in winter.

### Environmental predictors for coral growth

The environmental predictors of calcification varied by era (Table 2). In modern coral, the calcification rate was negatively correlated with warming SST and increasing Ba/Ca peaks. In fossil corals, no predictors were selected for the calcification rate (Table 2).

**Table 2:** Statistical model parameter estimates of modern and fossil corals at Nagura Bay.

Sample	Responseparameter	Explanatory parameters	AIC	Parameters	Slope	Error ofslope
Modern	Calcification rate	**Annual SST+Ba/Ca peak**	**9.2**	AnnualSST	−0.77	0.32
		Annual SST+Ba/Ca peak+Prep.	10	Ba/Capeak	−1.78	0.07
		DHM+Annual SST+Ba/Ca peak	10.6			
	Extension rate	**Annual SST+Ba/Ca peak**	−**7.3**	AnnualSST	−0.40	0.17
		DHM+Ba/Ca peak	−7.2	Ba/Capeak	−0.82	0.36
		DHM+Annual SST+Ba/Ca peak	−6.7			
	Skeletal density	**Annual SST+Ba/Ca** **peak+Prep.**	−**26.4**	AnnualSST	−0.25	0.18
		Annual SST+Ba/Ca peak	−25.6	Ba/Capeak	−0.62	0.10
		DHM+Annual SST+Ba/Capeak+Prep.	−25.2	Prep.	9.27E-05	6.25E-05
1.2 kyr BP	Calcification rate	**None**	**4.6**		–	
		Annual SST	6.3			
		DHM+Annual SST	6.3			
	Extension rate	**None**	−**6.9**		–	
		DHM+Annual SST	−6			
		DHM	−5.5			
	Skeletal density	**DHM**	−**22.6**	DHM	0.03	0.02
		Minimum SST	−21.9			
		Annual SST	−21.6			
3.5 kyr BP	Calcification rate	**None**	**59**		–	
		Annual SST	59.8			
		DHM	60.7			
	Extension rate	**DHM**	**33.6**	DHM	−0.07	0.07
		Annual SST	34.4			
		Minimum SST	35.3			
	Skeletal density	**DHM**	−**37.7**	DHM	0.05	0.02
		DHM+Annual SST	−38.6			
		Annual SST	−38.5			

Bold text indicates the selected models. Prep: precipitation.

## Discussion

### Reconstruction of the Holocene environmental history from 1.2 kyr BP and 3.5 kyr BP corals

The SST values reconstructed from the Sr/Ca values of the coral skeletons suggest that the modern characteristics of atmospheric circulation and the oceanographic setting at Nagura varied between the 1.2 and 3.5 kyr BP corals.

**Figure 9:**
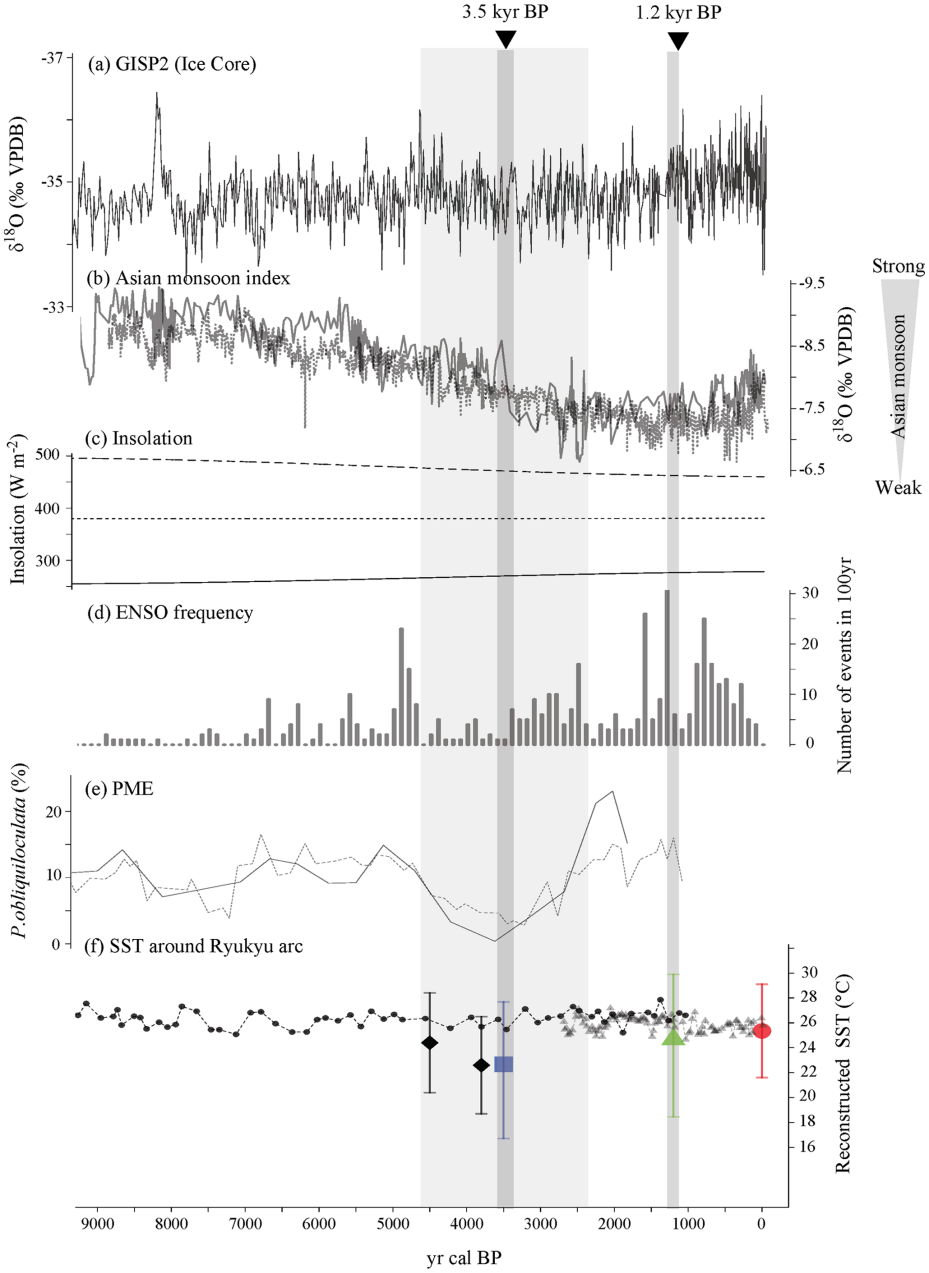
Climate changes during the late Holocene at approximately 25°N, Okinawa Trough. Two black triangles denote the date of fossil corals collected in this study. Vertical faint gray bar indicates the *Pulleniatina* Minimum Event (PME). (a) Time series of GRIP2 δ^18^O [61]. (b) δ^18^O records of Asian monsoon from Dogge Cave, China from [62] (solid gray line) and [63] (dashed gray line). (c) Averaged January (solid line), July (dashed line) and annual (dotted line) insolation at 25°N (W m^−2^) from [64]. (d) Number of ENSO events in 100 yr from [65]. (e) The timing of the PME in cores A7 from [68]. Y axis indicates the percent species abundance of *Pulleniatina obliquiloculata*. (f) Reconstructed SST from the present and previous studies. Red open circles, green triangles, and blue squares denote reconstructed SST data from this study. SST in each year is described as upper (averaged maximum SST), middle (annual SST), or lower (averaged minimum SST). Previous reconstructed SSTs were established by foraminifera *Globigerinoides ruber* Mg/Ca ratio in core 7 ([68]; circle), by TEX_86_ in IODP Hole 1202B ([69]; triangle), and by Sr/Ca ratio in *Porites* coral([70]; rhombus).

Figure 9 shows the environmental changes that occurred during the late Holocene, from 10,000 cal yr BP to the modern era. The GRIP2 δ^18^O values indicate conditions were stable from the early to mid-Holocene but suggest that these values were also highly varied (Figure 9 (a)) [61]. The Asia monsoon exhibited a decreasing trend (Figure 9 (b)) [62], [63], as did summer insolation, although winter insolation presented an increasing trend at 25°N (Figure 9 (c)) [64]. The El Niño-Southern Oscillation (ENSO) occurred with low frequency (less than 10 cycles during 100 yr) in the record from the 1.2 and 3.5 kyr BP corals (Figure 9 (d)) [65]. The late Holocene environment near the Okinawa Trough has been reconstructed from sediment cores and fossil corals [41], [66]–[68]. Between 4,600 and 2,300 cal yr BP, the *Pulleniatina* Minimum Event (PME) occurred [66], characterized by a low abundance of the tropical planktonic foraminifer *Pulleniatina obliquiloculata* (Figure 9 (e)). The paleo-SST values have been reconstructed by foraminifera Mg/Ca-thermometry in *Globigerinoides ruber* and by TEX_86_ and coral Sr/Ca thermometry in *Porites* sp. (Figure 9 (f)) [41], [69], [70].

Coral SST thermometry revealed cold events during the PME, as presented in this study and a few others. The Kuroshio Current and adjacent surface water masses experienced major changes from 4.5 to 3.0 ka (PME) in this region [66], which corresponds to the 3.5 kyr BP coral in this study. Previous studies suggested that the lower rates of surface transport in the Kuroshio Current, which changed in relation to El Niño-like conditions in the equatorial Pacific Ocean, resulted in the reduction of *Pulleniatina obliquiloculata* [67]. The cause of the PME is controversial. Some studies have indicated, based on foraminifera-based reconstructions of deep-sea sedimentary cores, that the PME is not likely to have been related to changes in SST and sea surface salinity (SSS) [67], [71]. For example, the SST was 25.9°C based on Mg/Ca thermometry [69]. However, cooler SSTs have been reported along the Okinawa Trough, based on δ^18^O and Sr/Ca-derived SST records in fossil coral skeletons from Kikai Island (3.4 and 3.7 kyr BP) and Kume Island (3.8 kyr BP), as well as in this study (Figure 9 (f)) [70], [72].

There are several typical problems associated with paleo-thermometry as calculated from Sr/Ca ratios in corals and Mg/Ca ratios in planktonic foraminifera [73]. The reconstructed glacial-Holocene shift in tropical SSTs, based on coral Sr/Ca (4–6°C) values, was larger than that indicated by the Mg/Ca of foraminifera (2–4°C) [74]–[83]. There are several possible reasons why coral-based SSTs might record cold SST events to a greater degree than foraminifera-based SSTs in the PME, such as differences in habitat depth. Massive corals live at depths shallower than several tens of meters; however, planktonic foraminifera live at depths of up to several hundred meters.

Another problem is the difference between the local SSTs of shallow (i.e., the coral reef) and deeper waters (i.e., the outer reef). The 3.5 kyr BP coral exists as a microatoll where sensitivity to the SST depends on tide cycle. However, the monthly SST gives an averaged value of the effect. The monthly SST of Shiraho Reef is warmer than that of Ishigaki Port in Ishigaki Island, with a maximum average SST difference between Shiraho Reef and Ishigaki Port (the outer reef) of approximately 1.0°C (Figure S1). Moreover, the uncertainty of the reconstructed SST in this study was approximately 1.0°C. Thus, a value in excess of ±2.0°C SST in comparison to the modern SST would indicate a significant difference between modern and paleo open sea SSTs. In light of these facts, the 3.5 kyr BP coral would give a cooler SST in the winter and thus cooler annual average in comparison to today.

If we assume the coral thermometry indicates the SST to a depth up to several tens of meters, the cause of the cool winter and annual SST values in the 3.5 kyr BP coral could be explained by reduced transport of the Kuroshio Current; this mechanism has in fact been previously put forward as an explanation [67]. At this site, the modern Kuroshio Current causes a winter SST that is warmer than the corresponding air temperature, where the average winter minimum SST is approximately 21.6°C and air temperature is approximately 18.6°C. The morphology of the 3.5 kyr BP coral is a microatoll; here, the surface is near the low tide level, and the SST is likely influenced by the air temperature. Furthermore, if transport reduction occurred due to the Kuroshio Current, the SST would be influenced to a greater degree by the air temperature. An additional problem is whether the historic air temperature was cooler than that today. The modern winter air temperature is similar to the winter SST within the limit of uncertainty at 3.5 kyr BP, thus obviating the need to explain that the comparatively cool SST identified in the 3.5 kyr BP is the result of the cooler air temperature of today. However, no quantitative measurement of the paleo air temperature has been recorded at this site to date. Further reconstruction of the air temperature would confirm the cause of the reduction in SST.

The annual and summer SSTs of the 1.2 kyr BP coral were not significantly cooler than that today. These SSTs are in good agreement with the findings of previous studies [69], [84]. This period (approximately 721–826 AD) is between the Sui-Tang Dynasty Warm Period (approximately 550–790 AD) and the Medieval Warm Period (approximately 900–1300 AD). The average SST during the Sui-Tang Dynasty Warm Period was 26.5°C based on Mg/Ca thermometry and 25.5°C based on TEX_86_ with no reported uncertainty [69], [70], which is similar to the annual SST obtained for the 1.2 kyr BP (24.7°C) with uncertainty. However, a significantly cooler than present SST occurred in winter for the 1.2 kyr BP. No reports to date have reported reduced transport of the Kuroshio Current in 1.2 kyr BP coral. Additionally, the fossil coral from 1.2 kyr BP is not a microatoll, and thus the cause of the winter SST for the 1.2 kyr BP is likely the result of cooler air temperature. However, to the best of our knowledge, few paleo-SSTs or air temperatures have been recorded in proximity to this site. Further studies on the paleo-SST and air temperature at of 1.2 kyr BP would elucidate the mechanism of cool winter SST events in this location.

In summary, although the cause of the cool SSTs in the 1.2 kyr BP and 3.5 kyr BP is unclear, we identified cooler winter and annual SSTs in the 3.5 kyr BP and a cooler winter SST in the 1.2 kyr BP.

### The source of the Ba/Ca ratios in the modern and fossil corals

Previous studies have indicated that Ba/Ca ratios in coral skeletons are linked to variations in SST, the seawater concentration of Ba, and the form of Ba sequestered in the coral skeletons [28]–[30], [35], [36], [85], [86]. The distribution of the activity coefficients, D* = (X_BaCO3_/X_CaCO3_)_coral_/([Ba^2+^]/[Ca^2+^])_seawater_ (X are mole fractions of aragonite, [Me] are mol concentration of aqueous solution), decreased from 10 to 50°C and was expressed by the equation D* = 2.42−0.03595T (°C) [87]. According to this equation, the coral Ba/Ca ratio should be higher in winter than in summer. Although significant annual periodicity and winter peaks of Ba/Ca were confirmed in the modern coral, no annual periodical peaks of Ba/Ca were observed in the fossil corals despite the natural conditions. We calculated seawater Ba from modern coral Ba/Ca using D* and averaged summer (28.9°C) and winter SSTs (21.6°C) for 1996–2008. The expected seawater Ba was from 24.5 to 25.3 nmol kg^−1^. The range was smaller than seasonal seawater Ba variation in Fusaki, which is near our site [88]. These findings suggest that SST dependency is not the main cause of the variation of Ba/Ca in modern and fossil corals.

Barium enrichment of seawater, which is mainly caused by upwelling and river discharge with sedimentation, would naturally increase the Ba/Ca of coral. Upwelling of nutrient-rich water has been previously reported to enhance seawater Ba concentration [89]. For example, the monthly seawater Ba concentrations at New Caledonia from April 1996 to May 1998 changed from 31.46 to 48.92 nmol kg^−1^ [38] as a result of upwelling generated by strong winds. Winter upwelling also has been related to zonal winds at higher latitudes in Japan. A previous study reported that winter coral Ba/Ca is strongly correlated with upwelling in the northern habitable limit of Japan [36]. Seawater Ba concentrations in the winter and spring seasons (32–36 nmol kg^−1^) were similar to in the summer season (25–34 nmol kg^−1^) at Fusaki (outer reef), and Inoda and Akaishi had seawater characteristics that were similar to that at the outer reef condition that at the outer reef at Ishigaki Island (Figure 1 (b)) [88]. However, the seawater Ba range at each site was smaller than that obtained by Montaggioni et al. [38]. This indicates that there is little proof supporting winter upwelling at this site. In the present study, coral samples were collected from either the nearshore area or near the estuary. Thus, the coral Ba/Ca values in this study are not likely to be influenced by upwelling.

The amount of sedimentation linked to land development would also cause significant annual periodicity in modern coral. In this study, high Ba/Ca peaks in modern coral were recorded mainly in the winter season. Osawa et al. observed the precipitation, water depth, flow velocity, and turbidity at Nagura Watershed on Ishigaki Island from August 2005 to November 2006 (N = 420) [60]. The authors reported that soil erosion from the bare land following the winter sugar cane harvest produced a maximum sediment yield and transport (water discharge) in the Nagura Watershed from February 22 to March 2 2006. The seawater Ba concentration was strongly influenced by river discharge in Nagura Bay [88]. The Ba concentration at the mouth of the Nagura River (70–88 nmol kg^−1^ in seawater; hereafter, we assume a water density of approximately 1.0 g cm^−3^) was approximately 2–3 times higher than at Fusaki (30–34 nmol kg^−1^ in seawater). Although the concentration of seawater Ba at Fusaki was relatively low, the concentration of Mn was comparatively higher (8.00–150 mmol kg^−1^ in seawater) than at either Inoda or Akaishi (4.00–52.0 nmol kg^−1^ in seawater) [88]. This suggests that manganese would be derived from the re-dissolution of accumulated terrestrial input, such as sediment from the Nagura River. Hence, the waters at Fusaki would be primarily influenced by river discharge even though a similar Ba concentration was found to that in outer reefs (Inoda and Akaishi). The modern coral was collected less than 2 km west of the mouth of the Nagura River, in Nagura Bay. The seawater conditions at Nagura should resemble those at Fusaki. Hence, the winter peaks of Ba/Ca in modern coral are likely influenced by the sediment concentration within the river discharge, which is in turn linked to land development; however, the fossil coral Ba/Ca peaks in various seasons are related to the sediment concentration in the river water, but predate modern land development.

Summer floods associated with the approach of typhoons do not reflect the periodic seasonal increase of Ba/Ca in the modern and fossil corals. In the present study, the minimum modern coral Ba/Ca values are recorded each year in the summer. Previous studies indicated that the seasonal wet season delivery of terrigenous Ba to the coastal ocean via rivers was the main source of the annual Ba/Ca variability in inshore coral, where few upwelling events occurred [29], [30], [90]. However, the summer seawater Ba concentration was lowest at Fusaki even though the water samples were collected 5 days after a typhoon approach [88]. This suggests that the residence time of Ba from the Nagura River in seawater is less than 5 days. It has been previously reported that a cyclone event with heavy rainfall along the coast of the Great Barrier Reef did not produce a significant discrete Ba/Ca ratio in coral skeletons [28]. Additionally, from a thermodynamic perspective, the uptake of Ba by coral is lowest in the summer [87]. Hence, we postulate that the small Ba/Ca peaks observed during summer were a result of the short residence time of Ba in seawater at this study site and the warm temperature of the water.

The existence of various compounds (organic matter or BaSO_4_) and forms (pre-existing surface, occluded in skeleton, or detrital contamination) of Ba in coral skeletons have been discussed [e.g., 37,86,91]. The organic matter in some non-lattice bound phases elevates coral Ba/Ca but is liable to decay with time [37], [92]. In the present study, because the fossil coral contained some variation in the Ba/Ca with the time series, the Ba is most likely trapped in the carbonate lattice. Well-preserved fossil coral skeletons show little sign of the influence of secondary aragonite deposits. Pretreatment of the powdered samples with Milli-Q water in this study also removed any pre-existing surface detritus. Although BaSO_4_ content can yield an additional potential error in estimating the Ba/Ca values of fossil corals [86], we did not confirm the existence of BaSO_4_ in the XRD analyses. Thus, our coral skeletons provide a record of the primary Ba/Ca variation.

In light of these facts, the best explanation for the annual and inter-annual variations in the Ba content of the coral seems to be the sediment concentration within the river runoff. In particular, the recent land development produces significant annual periodicity in the Ba/Ca of modern coral. Nagura Bay is influenced by the flow of red soil from the land, which increases the sedimentation and nutrient levels [42]. Hence, we treat the Ba/Ca values in coral skeletons as proxies of both sedimentation and nutrient level.

### Influence of land development on massive coral calcification

**Figure 10:**
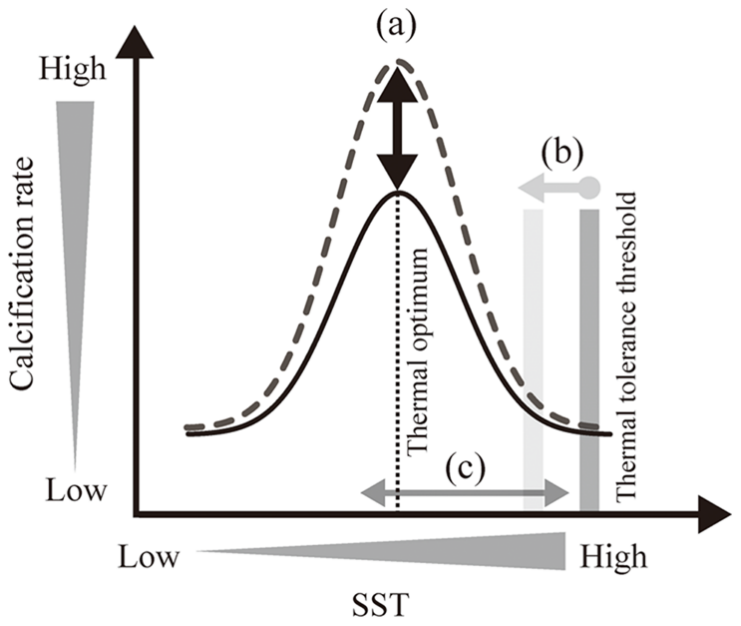
Schematic model of impact on coral calcification rate by land development. ( a) Fluctuation of absolute coral calcification rate is controlled by river input, which is related to land development in winter due to sugar cane harvest, as suggested by the present study. (b) Reduction of thermal tolerance threshold [9], [10]. (c) SST over the thermal optimum [16], [18].

In and around Nagura Bay (Figures 5 and S4), a clear reduction of coral calcification has occurred since the 1990s, which has also been reported for the Great Barrier Reef [15], [18], [21], Thailand [93], the Red Sea [20] and Panama [94]. Lough and Barnes noted that long-term average calcification rates in *Porites* along a latitudinal gradient increased with annual SST warming [95]. However, Carricart-Ganivet et al. and Castillo et al. [16], [19] also reported that recent massive coral growths were negatively correlated to warming SSTs. This tendency toward thermal sensitivity was also seen in our study (Table 2). In [9], the authors proposed that chronic local (anthropogenic) stress would alter coral physiology, reducing the coral’s thermal tolerance threshold (Figure 10 (b)). However, our study showed that changes in modern massive coral physiology due to anthropogenic stress from land development would not be limited solely to a reduction in thermal tolerance threshold. We therefore suggest that the impact of land development in winter due to sugar cane harvest would dictate the absolute coral calcification rate of the year (Figure 10 (a)).

Skeletal density in modern massive coral at Todoroki tended to increase from inshore to offshore (Figure 4), corresponding to spatial gradients of δ^15^N in microalgae as reported by Umezawa et al. [96], who suggest that terrestrial loading via rivers influences trends in δ^15^N in microalgae at Todoroki. As our *Porites* corals of TR 1 and TR 2 at Todoroki (Figure 1 (d)) were located near the site reported by Umezawa et al. [96], we considered our sites to also be influenced by terrestrial input via river input. Red soil and nutrient runoff linked to land development additionally induces environmental perturbations in shallow-water reef ecosystems at the Todoroki River as well [97]. Risk and Sammarco [98] suggested that nutrients, especially phosphate, act as inhibitors of calcification in nearshore waters, and other lab and field studies have found that under phosphate-enriched conditions, coral skeletons display increased porosity and decreased density [99], [100]. Therefore, coral skeletal density is likely to be impacted by the land development at Todoroki through river input containing red soil and nutrients.

The relationship between extension rate and distance from the coast at Todoroki also implies the influence of land development on coral growth. In general, an increase in extension rate with equal or lower deposition of calcium carbonate results in decreased skeletal density [101]. Edinger et al. [102] suggested that differences in density were directly related to changes in coral metabolism, driven by increasing sedimentation and nutrient levels on the polluted reefs. For example, low skeletal density with a high extension rate could be a possible indicator of eutrophication. We therefore propose that, spatially, land development would reduce only skeletal density at Todoroki (Figure 4).

Nagura is also influenced by these land development effects [42], [60], as supported by the periodical Ba/Ca ratio in modern coral skeletons (Figure 6). Furthermore, modern coral calcification declined with increasing Ba/Ca peaks in winter (Figure 8), which suggests that land development in winter due to sugar cane harvest influences calcification rate. Temporally, coral skeletal density was positively correlated with extension rate at Nagura (Figure 3), which is an unusual trend suggested by previous studies [95], [101]. Thus, these results indicate that the modern massive coral growth at Nagura was affected by land development in winter due to sugar cane harvest.

In the present study, SST had a negative effect on modern coral calcification rate at Nagura, which was not explained with the model of reduction in thermal tolerance threshold and/or recent SST over the coral thermal optimum (maximum calcification rate) suggested by previous studies (Figure 10 (b and c), Table 2). Our result was not consistent with the reduction in coral thermal tolerance threshold as a result of chronic anthropogenic stress (Figure 10 (b)) [9], [10], because Ba/Ca peaks in winter but not DHM, were selected as a parameter for coral calcification in this study.

The recent SST over coral thermal optimum also does not explain the negative thermal sensitivity (Figure 10 (c)). The previous studies suggested that the coral thermal optimum occurred in the warmest months where these authors collected their corals [103], [104]. Assuming a Gaussian-like function, Marshall and Clode reported that the thermal optimum was approximately 25°C [103]. The SST was nearly the same as the mean SST of the warmest months on Heron Island. Cooper et al. also suggested that a mean annual SST of 26.7°C was the thermal optimum for *Porites* corals in nearshore regions of the northern Great Barrier Reef [105]. However, Carricart-Ganivet et al. showed that *Porites* coral calcification decreased with warming SSTs of 25.9–26.5°C in the central Great Barrier Reef [23]. In our study site, annual SST ranged from 25.0°C to 26.1°C, which is below the thermal optimum and reduction range of SST in *Porites* corals. Therefore, assuming that the thermal optimum for *Porites* corals is roughly 26–26.7°C, we cannot explain the negative thermal sensitivity of calcification rate at Nagura with the model of recent SST over the thermal optimum (Figure 10 (c)). Factors other than SST likely influence their thermal sensitivity.

Thus, our results of geochemical signals and growth characteristics in modern and fossil corals at Nagura suggest that temporally, the impact of land development in winter due to sugar cane harvest negatively influences massive coral physiology and controls the absolute coral calcification rate, which induces negative thermal sensitivity in coral calcification rate (Figure 10 (a)).

Fossil coral data support the influence of land development to thermal sensitivity in modern coral. To estimate the thermal stress for coral growth, we define “growth stress year,” which was selected based on the following set of conditions, (i) reduced calcification in a given year in comparison to the previous year, (ii) increased calcification in the following year, and (iii) the occurrence of a bleaching event in that year. As a result, in modern coral, growth stress years were detected in 1998 and 2001 (Figure 5). Condition (i) is defined in a growth stress year as calcification reduced by less than −22.9% from the previous year in modern coral (Figure 5). Next, we applied the growth stress year to fossil corals (Figure 5). Growth stress year is not related to DHM in the 1.2 kyr BP coral. In the 3.5 kyr BP coral, only 3 occurrences of growth stress years from the set of 8 data points were related to DHM. Fossil corals also experienced higher rates and frequencies of DHM than modern corals. However, no reductions in the calcification rate or scars appeared at high rates of DHM in the fossil corals. This result agrees with the previous mid-century studies conducted at the Mesoamerican Coral Reef, which did not exhibit bleaching events despite high thermal stress until 1978 [7], [9]. However, SST was not the ultimate parameter controlling the calcification rate in fossil corals at Nagura in this study, although Lough and Barnes suggested from sclerochronological analysis of *Porites* corals at other sites that it was [95]. Some other studies have reported similar conclusions. For example, in studies of the fringing reefs of the central Caribbean coast of Panama and the back reefs of Belize, the annual skeletal extension for *Siderastrea siderea* was not found to be correlated with the measured environmental variables, including SST [14], [19], [106]. However, a few investigations have been conducted on fossil coral growth with respect to environmental changes. Recent geochemical analysis of coral skeletons has reconstructed SST (Sr/Ca, δ^18^O), SSS (δ^18^O), nutrient environment (Ba/Ca, P/Ca, δ^15^N), sediment (Ba/Ca, luminescence), and pH (δ^11^B) values [29], [73], [107]–[112]. Further investigation of the annual skeletal banding with geochemical signals in relation to environmental factors may further support our results suggesting the influence of land development on coral calcification rates.

The genus- or species-specific thermal optimum is an important factor for determining calcification rate but is still under discussion. Wórum et al. suggested that the mean SST of the warmest months in the Mexican Caribbean (28.8°C) is equivalent to the thermal optimum of *Montastraea* sp. [104]. Carricart-Ganivet suggested species-specific thermal responses in *M. annualaris*, and analyzed the relationships between SST and annual growth characteristics in the Caribbean Sea and Gulf of Mexico. While the SST range was different for the two locations, (Caribbean Sea 25.8–29.7°C, 1970–1997, 6 sites; Gulf of Mexico 22.8–29.8°C, 1970–1997, 6 sites), the coral’s calcification responses to changes in SST were similar [113]. On the other hand, Marshall and Clode reported that the thermal optimum was similar in the zooxanthellate *Galaxea fascicularis* and the azooxanthellate *Dendrophyllia* sp. [103].

Analysis of modern and Holocene *Porites* coral growth using seasonal geochemical signals (Sr/Ca and Ba/Ca ratios) at Nagura and Todoroki provides a new insight into coral growth as it is impacted by land development. At Nagura, compared to modern SST, the winter SST was cooler by 2.8°C in 1.2 cal kyr BP and the annual and winter SSTs in 3.5 cal kyr BP were cooler by 2.6°C and 4.6°C, respectively. Annual periodicity of coral Ba/Ca ratios was found only in the modern coral, which is likely linked to river discharge and land development. Although Holocene SST around the Okinawa Trough remain to be resolved, due to the fact that the paleo-SSTs reconstructed by geochemical analyses of coral and foraminifera differed, we find that the Sr/Ca (paleo-SST) and Ba/Ca (paleo-input of sediment and nutrients) ratios would not have a negative impact on fossil *Porites* coral calcification at Nagura. We also report negative thermal sensitivity for calcification rate as a result of land development in winter due to sugar cane harvest at Nagura. Our results suggest that modern *Porites* corals stressed by land development ultimately may be more vulnerable to recent ocean warming. However, we were unable to identify the cause of the physiological mechanisms of negative thermal sensitivity of coral calcification. Comparisons of coral calcification and historical land development information could help to elucidate the mechanism of this thermal sensitivity. Further observational and experimental investigations will be useful for verifying our hypothesis that changing thermal sensitivity for calcification rate is a specifically modern phenomenon. Understanding the influence of anthropogenic stress will provide additional information with respect to establishing an effective coral conservation plan for the future.

## Supporting Information

Figure S1 **Observed monthly sea surface temperature (SST; black line) and air temperature (gray line).** (a) wind speed (black line) and (b) number of typhoon approaches (gray bars) at Ishigaki Island from 1996 to 2008.
(TIF)

Figure S2 **Comparison of** ***in situ*** **average monthly SST for Shiraho Reef (black line) and Ishigaki Port (gray line), with monthly SST as reported by Integrated Global Ocean Services System Products Bulletin (black dotted line) and the average monthly temperature (gray dotted line) at Ishigaki Island, Japan, from July 2002 to February 2006.**
(TIF)

Figure S3 **Relationship between SST for Ishigaki Port and Shiraho Reef.** Regression line is shown where there is a statistically significant link.
(TIF)

Figure S4 **Average annual extension anomalies, 1990–2008, for modern corals from Nagura Bay and TE** [**59**] **.** Regression lines are shown where there is a statistically significant link.
(TIF)

Figure S5 **Regression between Sr/Ca and (bi-monthly average) SST data sets from Ishigaki Port.** Dashed line denotes the 1σ value.
(TIF)

Figure S6 **The Blackman-Turkey power spectra for (a-1) modern SST (gray line), (b) 1.2 kyr BP and (c) 3.5 kyr BP coral Sr/Ca (gray line) and Ba/Ca (black lines) ratios.** In (c), dotted lines and solid lines indicate years 1–11 and 14–38, respectively. (a-2) and (a-3) indicate the coherency and phase, respectively, of modern coral SST and Ba/Ca ratio. All vertical and horizontal error bars indicate 90% confidence intervals.
(TIF)

Figure S7 **Timing of Ba/Ca peaks, indicated as percentages, in (a) modern, (b) 1.2 kyr BP, and (c) 3.5 kyr BP corals.**
(TIF)
