# Supplementary figures and images for: Influence of Land Development on Holocene Porites Coral Calcification at Nagura Bay, Ishigaki Island, Japan
Source: PLoS One. 2014 Feb 24;9(2):e88790. doi: 10.1371/journal.pone.0088790 (PMC3933341; doi:10.1371/journal.pone.0088790)

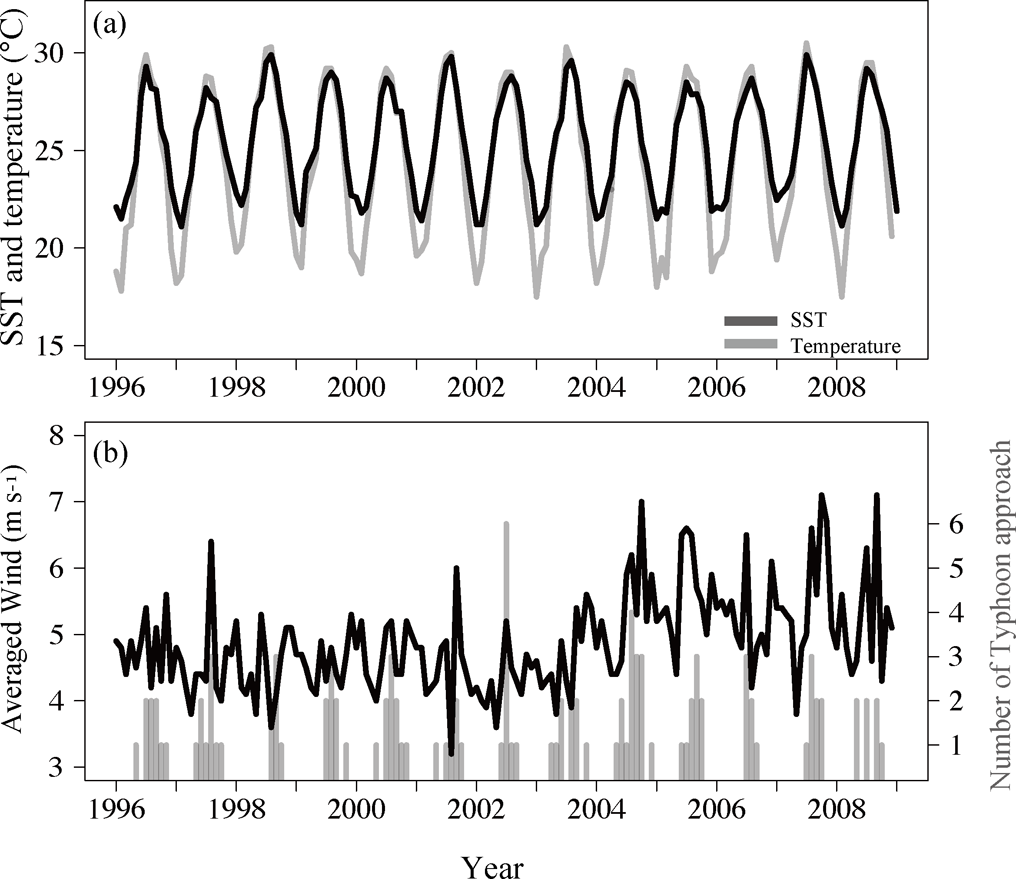

Supplement: Figure S1 — Observed monthly sea surface temperature (SST; black line) and air temperature (gray line). (a) wind speed (black line) and (b) number of typhoon approaches (gray bars) at Ishigaki Island from 1996 to 2008. (TIF) [file pone.0088790.s001.tif]

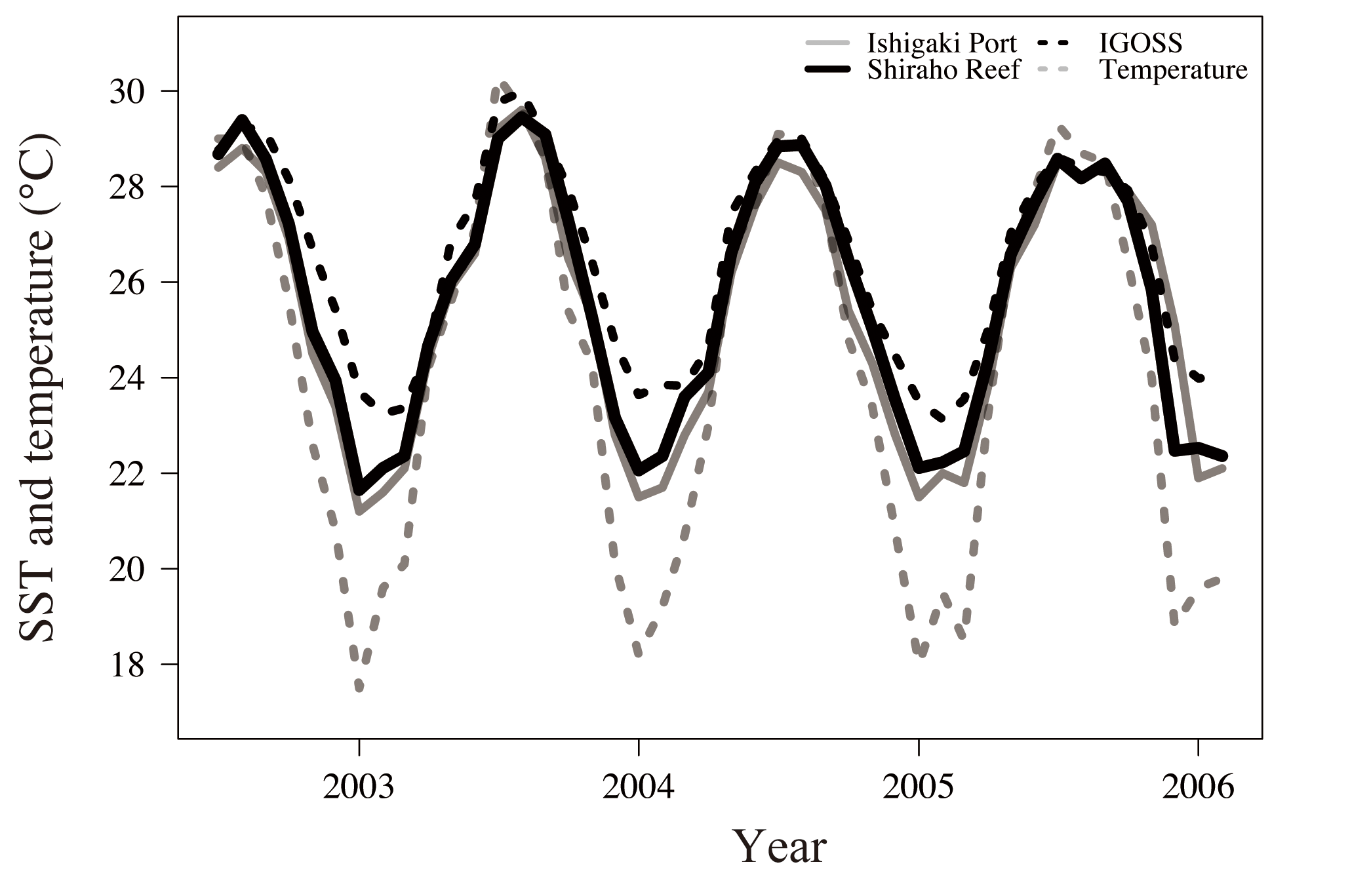

Supplement: Figure S2 — Comparison of in situ average monthly SST for Shiraho Reef (black line) and Ishigaki Port (gray line), with monthly SST as reported by Integrated Global Ocean Services System Products Bulletin (black dotted line) and the average monthly temperature (gray dotted line) at Ishigaki Island, Japan, from July 2002 to February 2006. (TIF) [file pone.0088790.s002.tif]

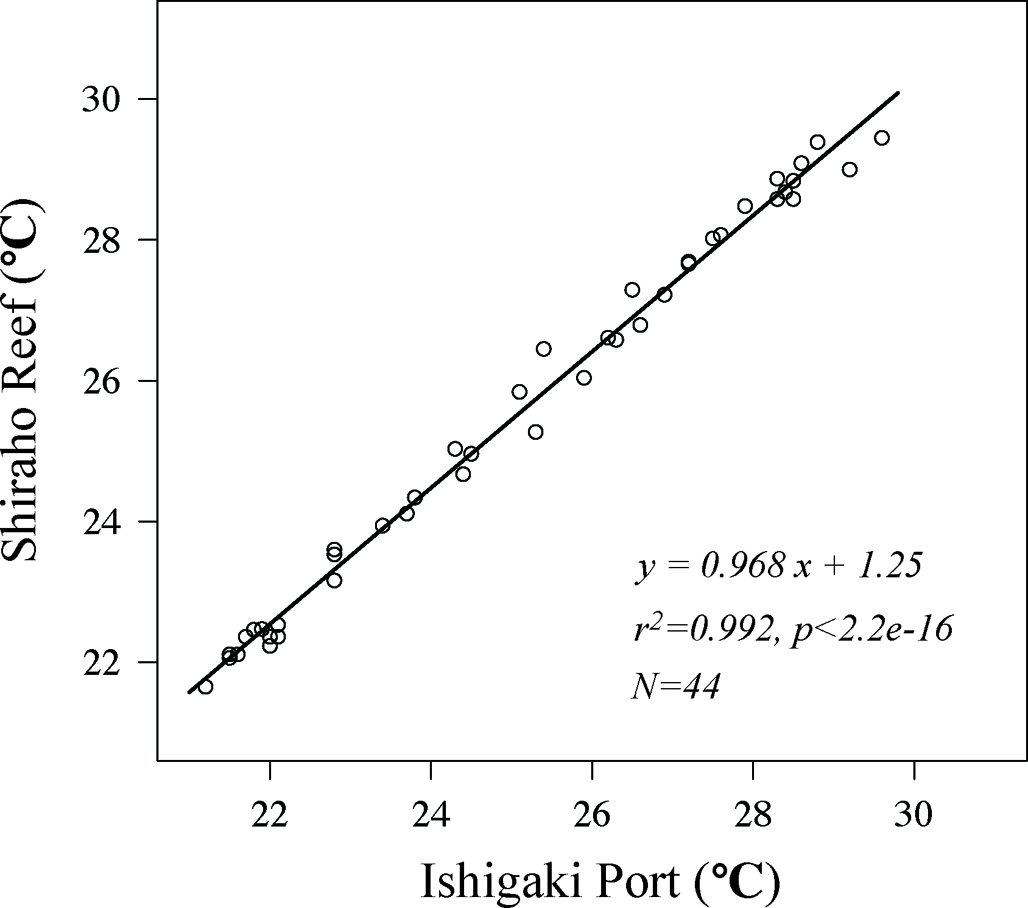

Supplement: Figure S3 — Relationship between SST for Ishigaki Port and Shiraho Reef. Regression line is shown where there is a statistically significant link. (TIF) [file pone.0088790.s003.tif]

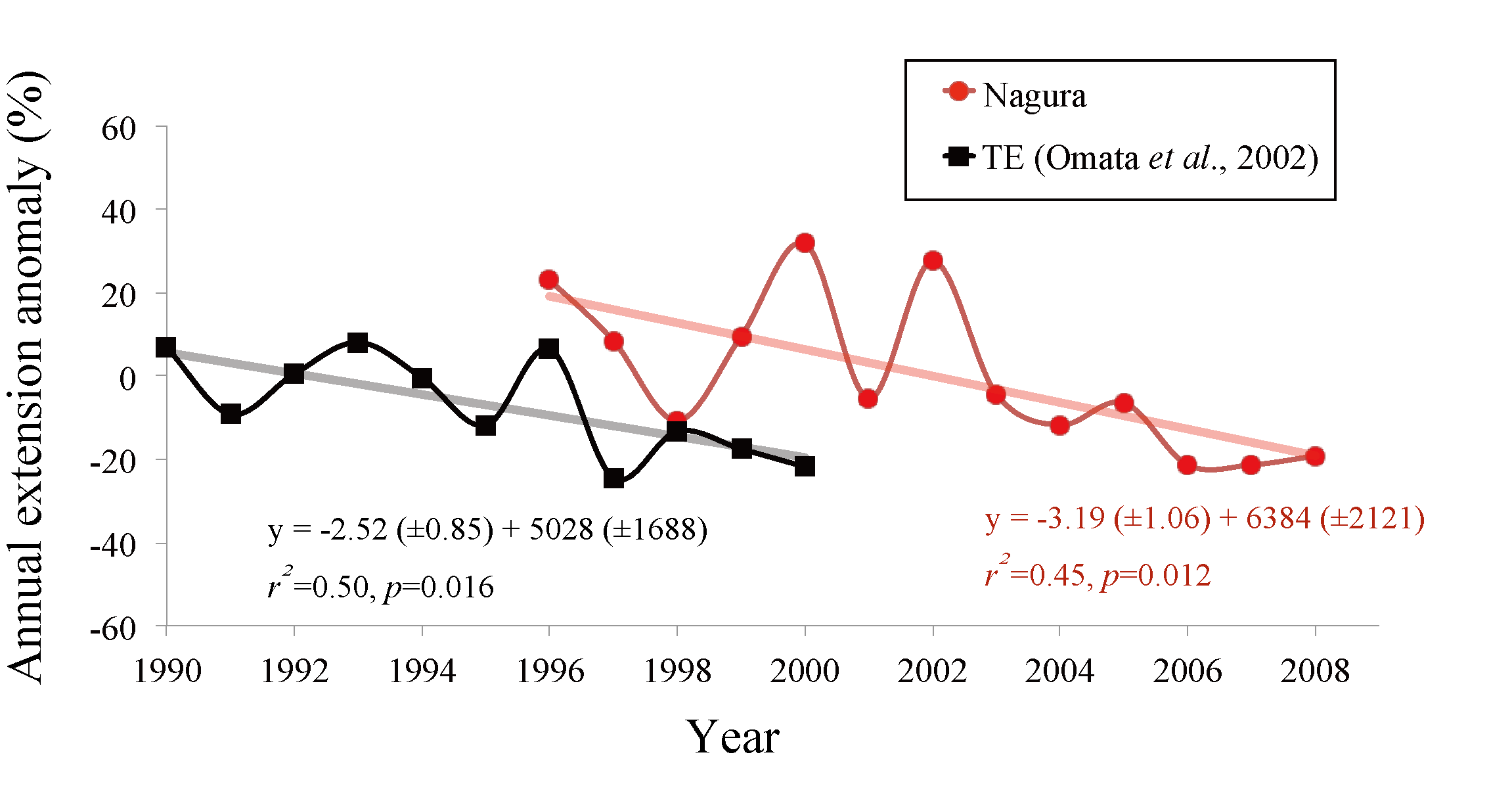

Supplement: Figure S4 — Average annual extension anomalies, 1990–2008, for modern corals from Nagura Bay and TE [59] . Regression lines are shown where there is a statistically significant link. (TIF) [file pone.0088790.s004.tif]

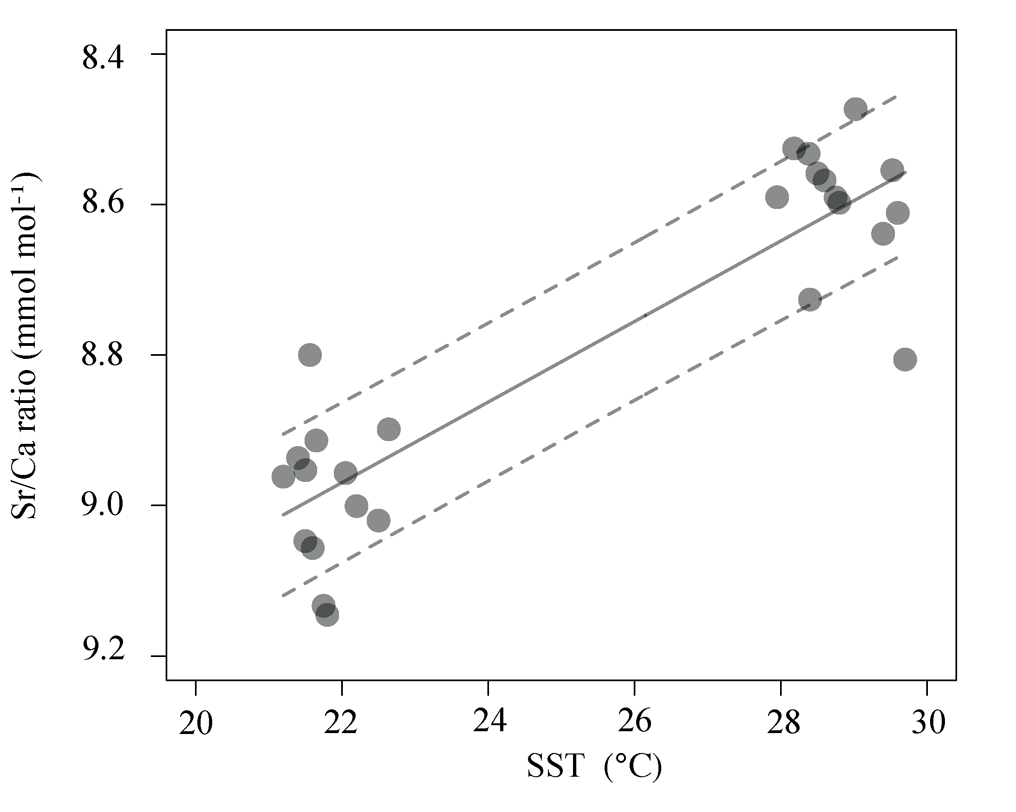

Supplement: Figure S5 — Regression between Sr/Ca and (bi-monthly average) SST data sets from Ishigaki Port. Dashed line denotes the 1σ value. (TIF) [file pone.0088790.s005.tif]

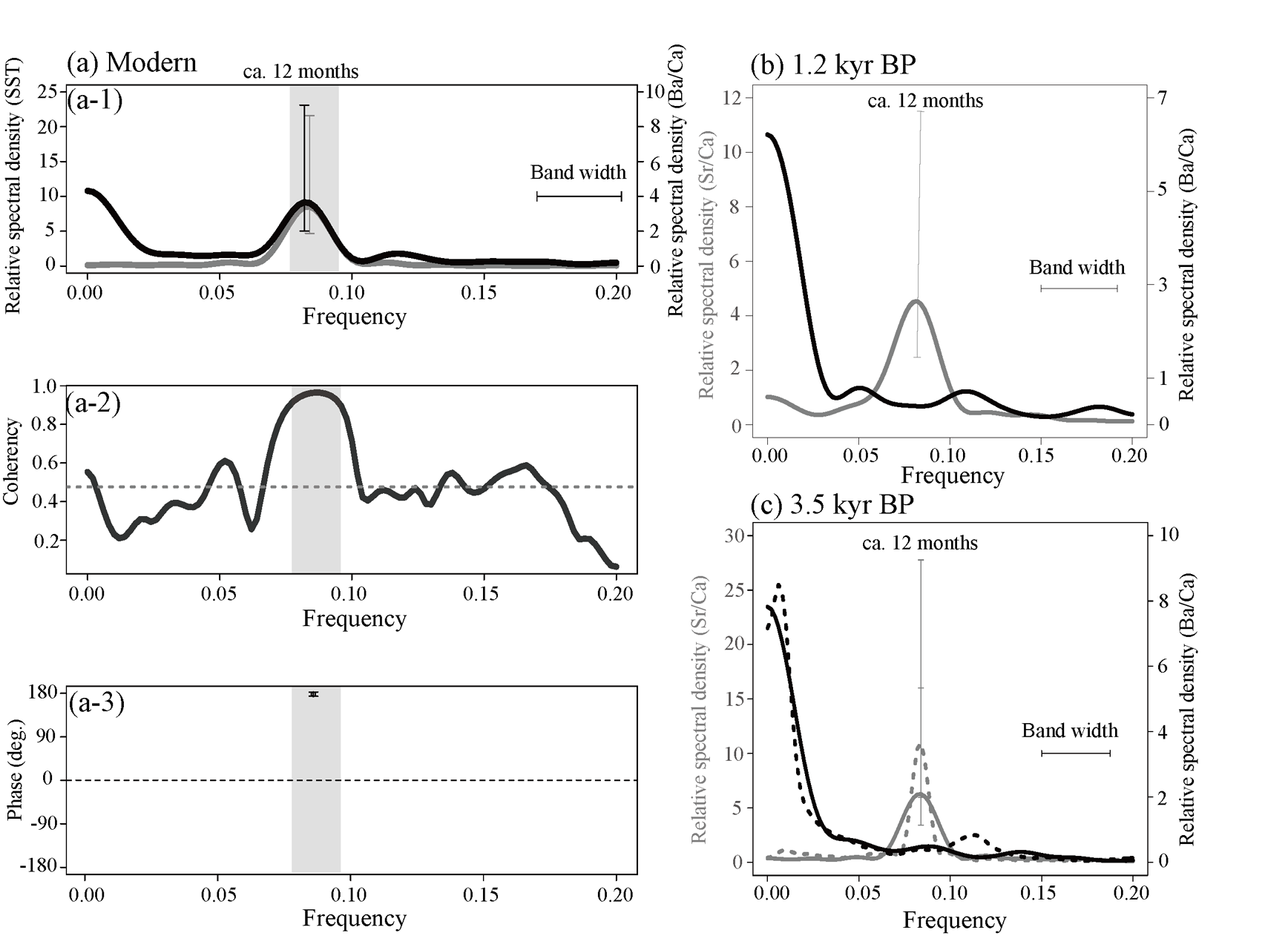

Supplement: Figure S6 — The Blackman-Turkey power spectra for (a-1) modern SST (gray line), (b) 1.2 kyr BP and (c) 3.5 kyr BP coral Sr/Ca (gray line) and Ba/Ca (black lines) ratios. In (c), dotted lines and solid lines indicate years 1–11 and 14–38, respectively. (a-2) and (a-3) indicate the coherency and phase, respectively, of modern coral SST and Ba/Ca ratio. All vertical and horizontal error bars indicate 90% confidence intervals. (TIF) [file pone.0088790.s006.tif]

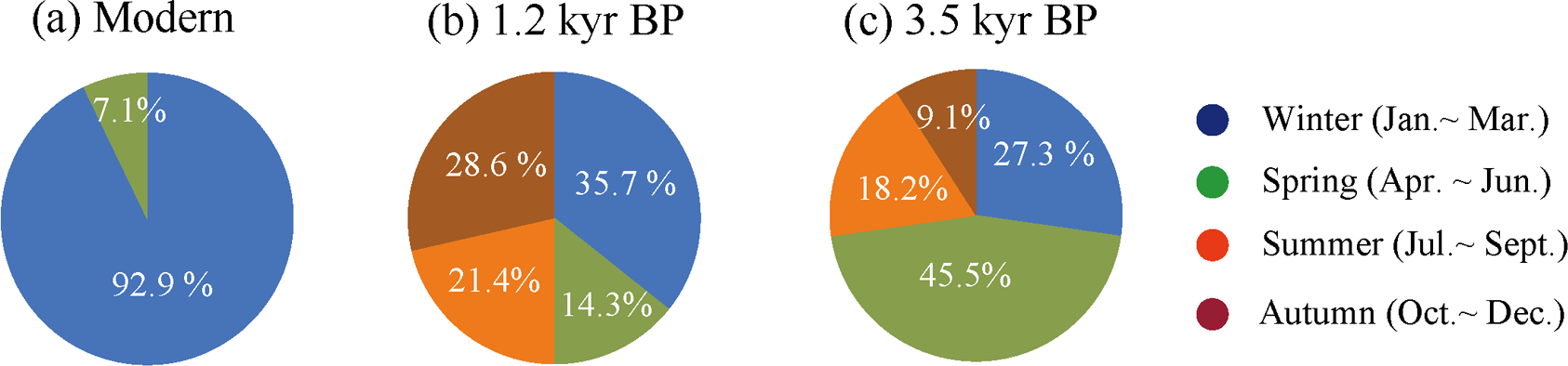

Supplement: Figure S7 — Timing of Ba/Ca peaks, indicated as percentages, in (a) modern, (b) 1.2 kyr BP, and (c) 3.5 kyr BP corals. (TIF) [file pone.0088790.s007.tif]
